# Bacterial TIR-based immune systems sense phage capsids to initiate defense

**DOI:** 10.1038/s41564-025-02150-0

**Published:** 2025-10-24

**Authors:** Cameron G. Roberts, Chloe B. Fishman, Zhiying Zhang, Dalton V. Banh, Dinshaw J. Patel, Luciano A. Marraffini

**Affiliations:** 1 Laboratory of Bacteriology, The Rockefeller University, New York, NY USA; 2 Structural Biology Program, Memorial Sloan Kettering Cancer Center, New York, NY USA; 3 Howard Hughes Medical Institute, The Rockefeller University, New York, NY USA

**Keywords:** Bacteriophages, Bacteria

## Abstract

Thoeris systems use proteins with Toll/interleukin-1 receptor domains to protect prokaryotes from phage infection through the synthesis of a cyclic adenosine diphosphate ribose signalling molecule, which activates an effector that depletes the host of the essential metabolite NAD^+^ to limit viral propagation. How infection is recognized during Thoeris immunity is not known. Here we investigate the staphylococcal Thoeris defense system, ThsA–B1–B2, and found that, upon infection, the major capsid proteins of *Siphoviridae* phages from serogroup B, but not A, form a complex with ThsB1 and ThsB2 to activate Thoeris defense. Thoeris cyclases from *Streptococcus* also recognize major capsid proteins. Our results suggest that the accumulation of capsid mutations that enable avoidance of Thoeris recognition may be an important evolutionary force behind the structural diversity of prokaryotic viruses. More broadly, given that some mammalian immune pathways contain Toll/interleukin-1 receptor domains that recognize viral structures, our findings highlight a conserved mechanism of innate antiviral immunity.

## Abstract (web summary)

After sensing capsids, the Thoeris antiphage defense system triggers a cascade that leads to NAD^+^ cleavage and cell arrest, which are dynamics that are mirrored in some mammalian immune pathways.

## Main

Recent discoveries have revealed bacterial antiphage defense systems that are structural and functional analogues to innate immunity in animals and plants^1^. One such system, Thoeris, uses proteins with Toll/interleukin-1 receptor (TIR) domains—known in metazoans for forming scaffolds essential for Toll-like receptor (TLR) signalling^2^ and in plants for hydrolysing nicotinamide adenine dinucleotide (NAD^+^)^3,4^. Thoeris systems, found in ~4% of bacterial and archaeal genomes across nine phyla, fall into two types on the basis of gene content^5^. Type I systems include a TIR-domain sensor (ThsB) and an NAD^+^-degrading effector (ThsA) with a Sir2/TIR-associating LOG-Smf/DprA (STALD) NAD^+^-binding domain. Upon viral infection, ThsB synthesizes the cyclic nucleotide glycocyclic adenosine diphosphate ribose (gcADPR)^5–7^, which binds to the STALD domain of ThsA and induces a conformational change that activates NAD^+^ degradation by the Sir2 domain^5,8^. NAD^+^ depletion arrests the growth of the infected cells and prevents viral propagation, providing community-level immunity through the continued replication of the uninfected cells within the population^9^. How ThsB senses phage invasion, however, remains unknown. Here, we investigate this unanswered question in the bacterium *Staphylococcus aureus*.

## Results

### Thoeris provides antiphage protection in staphylococci

**Fig. 1:**
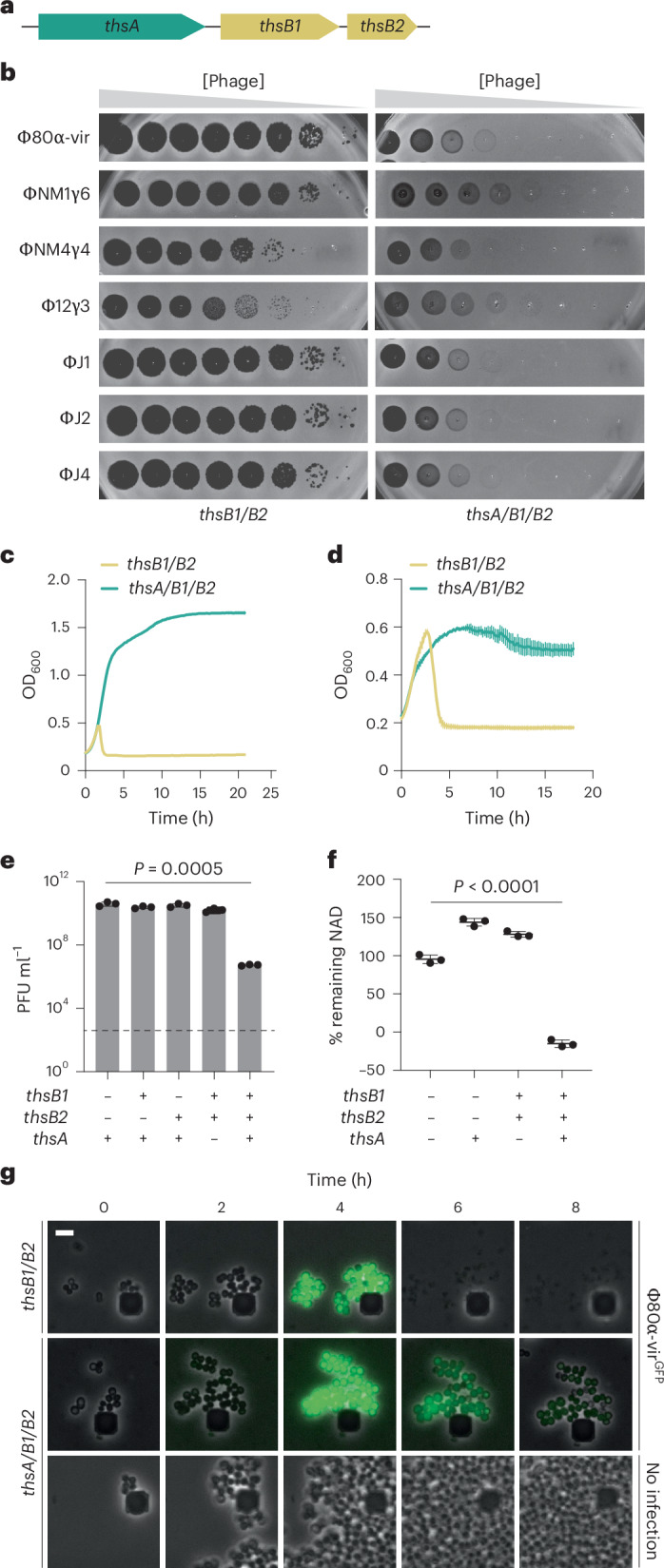
Thoeris provides antiphage protection in staphylococci. **a**, Schematic of the Thoeris operon present in the *Staphylococcus aureus* strain 08BA02176. The operon includes a *thsA* gene harbouring a STALD domain, and two *thsB* genes, *thsB1* and *thsB2*, that encode TIR domains. **b**, Tenfold serial dilutions of different staphylococcal phages on lawns of *S. aureus* RN4220 harbouring plasmids carrying either an incomplete (*thsB1/B2*) or full (*thsA/B1/B2*) Thoeris operon. **c**, Growth of *S. aureus* RN4220 harbouring plasmids carrying either an incomplete (*thsB1/B2*) or full (*thsA/B1/B2*) Thoeris operon, determined as the OD_600_ of the cultures after infection with Φ80α-vir at MOI 1. Mean ± s.d. of three biological replicates is reported. **d**, The same as in **c** but following the growth of lysogenic cultures after induction of the Φ80α prophage with MMC. **e**, Enumeration of PFU ml^−1^ after induction of the Φ80α prophage with MMC present in lysogens harbouring plasmids carrying different combinations of the *thsA*, *thsB1* and *thsB2* genes. Dashed line indicates the limit of detection. Mean ± s.d. of three biological replicates is reported; *P* value was obtained using an unpaired, two-tailed, *t*-test. **f**, Measure of percentage remaining NAD(H), calculated as the ratio of the concentration of NAD^+^ and NADH detected in staphylococci harbouring plasmids carrying different combinations of the *thsA*, *thsB1* and *thsB2* genes to the value detected in the absence of any of the *ths* genes, after induction of the Φ80α prophage with MMC. Mean ± s.d. of three biological replicates is reported; *P* value (<0.0001) was obtained using an unpaired, two-tailed, *t*-test comparing the group with no immunity with the group with full immunity. **g**, Fluorescence microscopy of *S. aureus* RN4220 harbouring plasmids carrying either an incomplete (*thsB1/B2*) or full (*thsA/B1/B2*) Thoeris operon. Images were taken every 2 h after infection with Φ80α-vir^GFP^ phage, up to 8 h. The images are representative of three independent experiments. Scale bar (top left), 1 µm.

**Extended Data Fig. 1:**
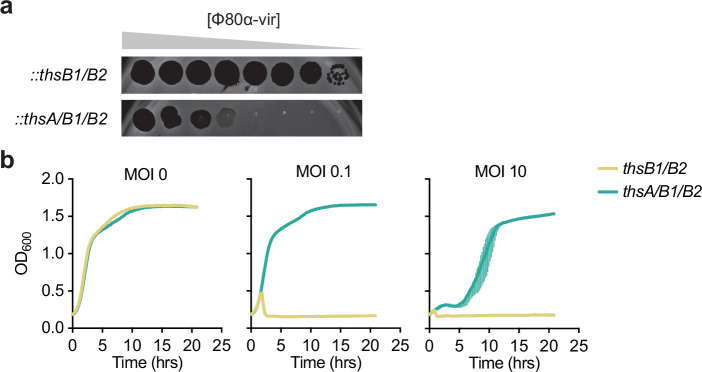
Characterization of Sau–Thoeris defense. **a)** Tenfold serial dilutions of Φ80α-vir on lawns of *S. aureus* RN4220 carrying either an incomplete (*thsB1/B2*) or full (*thsA/B1/B2*) Thoeris operon in the bacterial chromosome. **(b)** Growth of *S. aureus* RN4220 harboring plasmids carrying either an incomplete (*thsB1/B2*) or full (*thsA/B1/B2*) Thoeris operon, determined as the OD_600_ of the cultures after infection with Φ80α-vir at MOI 0, 0.1 or 10. Mean of +/- S.D. of three biological replicates is reported.

A search for Thoeris systems in staphylococci identified 32 unique but highly conserved operons (Supplementary Data 1). We focused on the type I system from *S. aureus* 08BA02176 (ref. ^10^) (hereafter Sau–Thoeris) composed of three genes (Fig. 1a): two TIR-domain sensors, ThsB1 and ThsB2, which are expected to synthesize gcADPR^5,6,8,11^ using NAD^+^ as substrate, and a gcADPR-dependent effector, ThsA, recently shown to degrade NAD^+^ to limit phage propagation^5,8^. We cloned the *thsA/B1/B2* operon into the staphylococcal vector pE194 (ref. ^12^) under the control of the P*spac* isopropyl-d-1-thiogalactopyranoside (IPTG)-inducible promoter for expression in the laboratory strain *S. aureus* RN4220 (ref. ^13^). Expression of *thsA/B1/B2*, but not *thsB1/B2* alone, reduced plaque formation for Φ80α-vir^14^, ΦNM1γ6 (ref. ^15^), ΦNM4γ4 (ref. ^16^), ΦJ1, ΦJ2, and ΦJ4 (ref. ^14^), but not for Φ12γ3 (ref. ^17^), staphylococcal phages (Fig. 1b, Extended Data Fig. 1a and Supplementary Fig. 1, which provides all the unedited images presented in this study). As reported previously^5^, Sau–Thoeris immunity supported the growth of infected cultures at low multiplicity of infection (MOI) (Fig. 1c and Extended Data Fig. 1b). Immunity was also activated after the induction of a Φ80α prophage (Fig. 1d,e). Quantification of total NAD (NAD^+^ and NADH, or NAD(H)) in cell lysates after Φ80α prophage induction showed a significant decrease only in the presence of the full *ths* operon (Fig. 1f). Finally, we visualized Sau–Thoeris defense at the cellular level using a modified Φ80α-vir phage that expresses GFP (Φ80α-vir^GFP^)^14^ and observed a decrease in green fluorescence of infected staphylococci without cell lysis, only upon expression of the full *ths* operon (Fig. 1g). We did not detect bacterial growth after 16 h, as opposed to the cultures that grew in liquid cultures within plate readers (Fig. 1c,d), probably owing to different aeration conditions in these assays. Altogether, these data show that, as previously reported for other systems^5,18^, Sau–Thoeris activation results in a depletion of NAD^+^ levels that inhibits viral propagation.

### The phage major head protein activates Thoeris in vivo

**Fig. 2:**
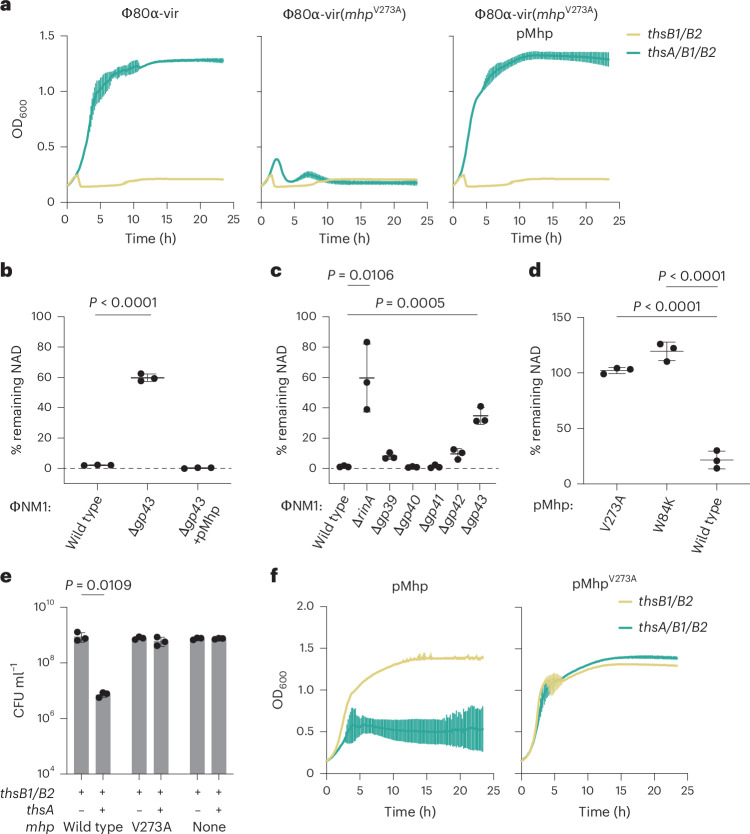
The phage Mhp activates Sau–Thoeris in vivo*.* **a**, Growth of *S. aureus* RN4220 harbouring plasmids carrying either an incomplete (*thsB1/B2*) or full (*thsA/B1/B2*) Thoeris operon in the absence or presence of a second plasmid expressing Mhp (not over-expressed), determined as the OD_600_ of the cultures after infection with Φ80α-vir or Φ80α-vir(*mhp*^V273A^) at MOI 1. Mean ± s.d. of three biological replicates is reported. **b**, Measure of percentage remaining NAD^+^/NADH (NAD), calculated as the ratio of the concentration of NAD^+^ and NADH detected in staphylococci harbouring a plasmid carrying a full (*thsA/B1/B2*) Thoeris operon to the value detected in the presence of an incomplete (*thsB1/B2*) system, after induction of the ΦNM1 prophage with MMC. Induced lysogens carried either wild type or mutant prophages with deletions in different genes involved in capsid formation. Mean ± s.d. of three biological replicates is reported; *P* values (*P* = 0.0106, *P* = 0.0005) were obtained using an unpaired, two-tailed, *t*-test comparing the wild type group with mutants. **c**, The same as in **b** after induction of wild type and Δ*gp43* ΦNM1 prophages, in the presence of a plasmid that expresses Mhp (*P* value <0.0001, unpaired, two-tailed, *t*-test). **d**, The same as in **b** but after IPTG induction of plasmid-encoded wild type, V273A, or W84K Mhp (*P* values <0.0001). **e**, Enumeration of CFU ml^−1^ after induction of the ΦNM1 prophage with MMC present in lysogens harbouring plasmids carrying either an incomplete (*thsB1/B2*) or full (*thsA/B1/B2*) Thoeris operon. Mean ± s.d. of three biological replicates is reported (*P* value <0.0001, unpaired, two-tailed, *t*-test). **f**, Growth of *S. aureus* RN4220 harbouring plasmids carrying either an incomplete (*thsB1/B2*) or full (*thsA/B1/B2*) Thoeris operon in the presence of a second plasmid expressing either wild type or V273A Mhp, determined as the OD_600_ of the cultures after addition of IPTG. Mean of ± s.d. of three biological replicates is reported.

**Extended Data Fig. 2:**
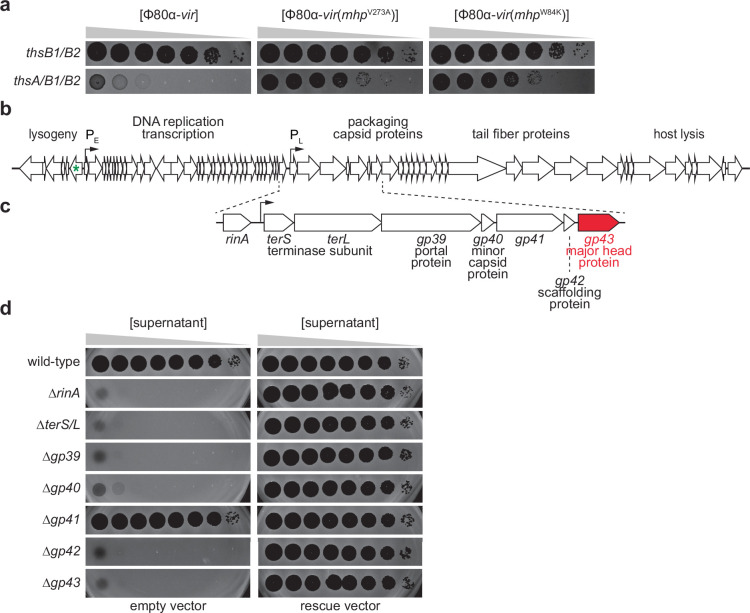
Mhp is required for Sau–Thoeris activation. **(a)** Tenfold serial dilutions of Φ80a-vir phage carrying wild type, V273A, W84K *mhp* alleles on lawns of *S. aureus* RN4220 harboring plasmids carrying either an incomplete (*thsB1/B2*) or full (*thsA/B1/B2*) Thoeris operon. **(b)** Schematic of ΦNM1 genome, with expansion of the operon for packaging the genome and assembly of phage particles. P_E_ and P_L_ are promoters responsible for the expression of early and late viral genes, respectively. The green asterisk indicates the nonsense mutation prevents ΦNM1 lysogeny, converting it into ΦNM1g6, a purely lytic phage. Regions of the genome involved in different stages of the viral lytic cycle are indicated. **(c)** Genes of the ΦNM1 genome required for capsid biogenesis. **(d)** Tenfold serial dilutions of lysates obtained after MMC induction of different ΦNM1 lysogens carrying deletions on genes involved in packaging the genome and assembly of phage particles on lawns of *S. aureus* RN4220 harboring an empty vector control, or rescue plasmids expressing the genes deleted in the prophages.

To investigate how the Sau–Thoeris response is triggered during phage infection, we isolated phages that overcome defense with the expectation that they would carry mutations in genes that are required for the activation of immunity. We sequenced the genomes of four phages that were able to form plaques in lawns of staphylococci expressing *thsA/B1/B2* and identified in all cases a missense mutation (V273A) in *gp47*, the gene encoding the major head protein (Mhp). The mutant phage lysed infected cultures (Fig. 2a) and formed plaques (Extended Data Fig. 2a) despite the presence of the Sau–Thoeris system. Induction of wild type ΦNM1 prophage^19^, but not of a mutant carrying a deletion of the gene encoding Mhp (*gp43*), led to a reduction of NAD(H) levels (Fig. 2b), a result that demonstrates the requirement of Mhp to trigger the Sau–Thoeris response. Both the absence of Mhp and the V273A mutation could be affecting Sau–Thoeris activation indirectly, by preventing the formation of a proper capsid structure. The ΦNM1 procapsid comprises 415 units of Mhp, 100–200 scaffolding proteins (*gp42*), a 12-unit portal complex (*gp39*), the terminase subunits (*terS/L*) responsible for viral DNA packaging into the procapsid to form a mature capsid^20^, and ~20 units of a minor head protein (*gp40*)^21^. This region of the ΦNM1 genome (Extended Data Fig. 2b,c) also harbours a small open reading frame of unknown function, *gp41*. With the exception of this gene, deletion of the structural genes from a ΦNM1 prophage, as well as *rinA*, which is required for the transcription of the phage structural genes^22^, abrogated the formation of viral particles upon induction, which was restored upon expression of the missing gene from a complementing plasmid (Extended Data Fig. 2d). Measurement of NAD(H) levels after mutant prophage induction revealed that only Δ*rinA* and Δ*gp43* mutants failed to activate Sau–Thoeris, a result that demonstrates that Mhp itself, and not a procapsid nor mature capsid, is necessary for activation.

To test if Mhp is sufficient to trigger Sau–Thoeris immunity, we expressed wild type and V273A Mhp using staphylococcal plasmid pC194 (ref. ^23^) under the control of the P*spac* promoter^13^ (pMhp), in the absence of phage infection. Expression of wild type, but not the V273A mutant, Mhp significantly reduced the cellular concentration of NAD(H) (Fig. 2d). As NAD^+^ depletion inhibits growth (Fig. 1g), we hypothesized that *mhp* expression in the presence of the *ths* operon should impair viability. Indeed, IPTG-induced expression of wild type, but not V273A, *mhp* significantly reduced colony-forming units (CFUs) (Fig. 2e) as well as growth in liquid media (Fig. 2f), only when the full operon was present (Fig. 2e). These findings demonstrate that the Mhp of ΦNM1 and Φ80α phages is both necessary and sufficient to activate Sau–Thoeris immunity.

### ThsB1, ThsB2 and Mhp form an active complex in vivo

**Fig. 3:**
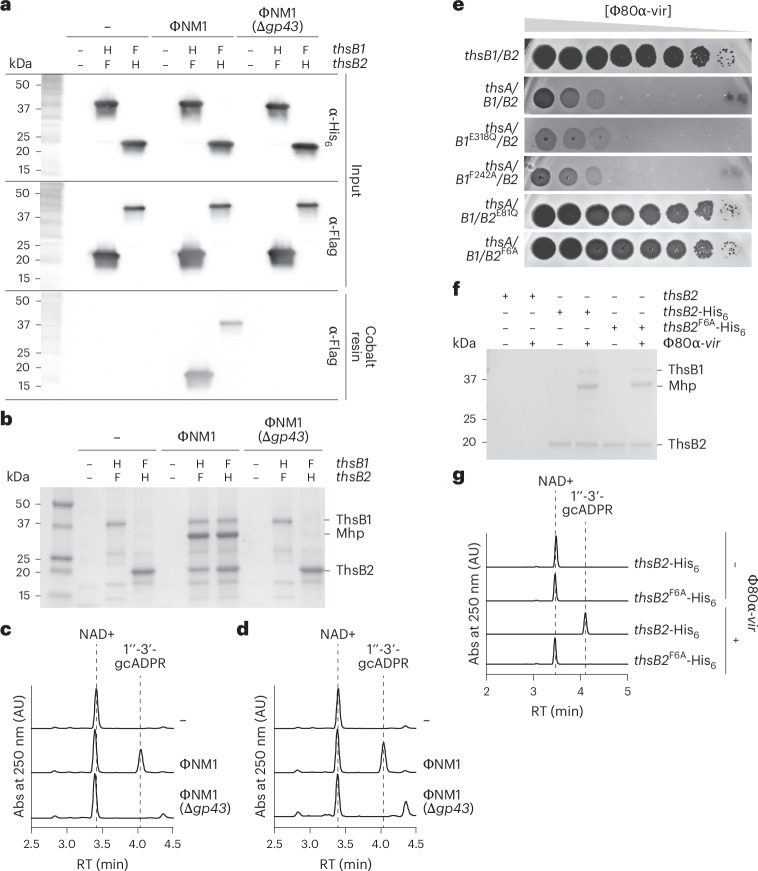
ThsB1, ThsB2 and Mhp form an active complex in vivo*.* **a**, Immunoblot analysis of proteins extracted from staphylococci expressing hexahystidyl- (H) or Flag- (F) tagged versions of ThsB1 or ThsB2, uninfected (−) or infected with wild type or Δ*gp43* ΦNM1 phage, either before (input) or after affinity chromatography using a cobalt resin. Proteins were separated by SDS–PAGE and electrotransferred to a polyvinylidene difluoride membrane. Tagged proteins were detected with antihexahystidyl (a-His) or anti-Flag (a-Flag) antibodies and chemiluminescence staining. **b**, Coomassie Blue-stained SDS–PAGE of proteins isolated after cobalt resin affinity chromatography in the experiment described in **a**. His_6_-ThsB1, 41.8 kDa; ThsB2-His_6_, 23.4 kDa; Mhp, 36.8 kDa. Protein molecular weight (kDa) markers are shown. **c**, HPLC analysis of the products resulting from the incubation of the proteins purified from staphylococci expressing His_6_-ThsB1 and ThsB2-Flag, uninduced or induced with wild type or Δ*gp43* ΦNM1 phage, with NAD^+^, using a cobalt resin. Retention times (RT) of reactants and products are marked with dashed lines. Absorbance (Abs) at 250 nm is reported. **d**, The same as in **c** but using proteins extracted from staphylococci expressing Flag-ThsB1 and ThsB2-His_6_. **e**, Tenfold serial dilutions of Φ80α-vir on lawns of *S. aureus* RN4220 harbouring plasmids carrying either an incomplete (*thsB1/B2*) or full (*thsA/B1/B2*) Thoeris operon carrying wild type or mutant versions of *thsB1* or *thsB2*. **f**, Coomassie Blue-stained SDS–PAGE of proteins isolated from staphylococci expressing ThsB1 with ThsB2, ThsB2-His_6_ or ThsB2^F6A^-His_6_, uninfected or infected with Φ80α-vir, after cobalt resin affinity chromatography. Protein molecular weight (kDa) markers are shown. **g**, HPLC analysis of the products resulting from the incubation of the proteins purified from staphylococci expressing ThsB1 with ThsB2-His_6_ or ThsB2^F6A^-His_6_, uninfected or infected with Φ80α-vir, with NAD^+^, using a cobalt resin. RT of reactants and products is marked with dashed lines. Absorbance (Abs) at 250 nm is reported.

**Extended Data Fig. 3:**
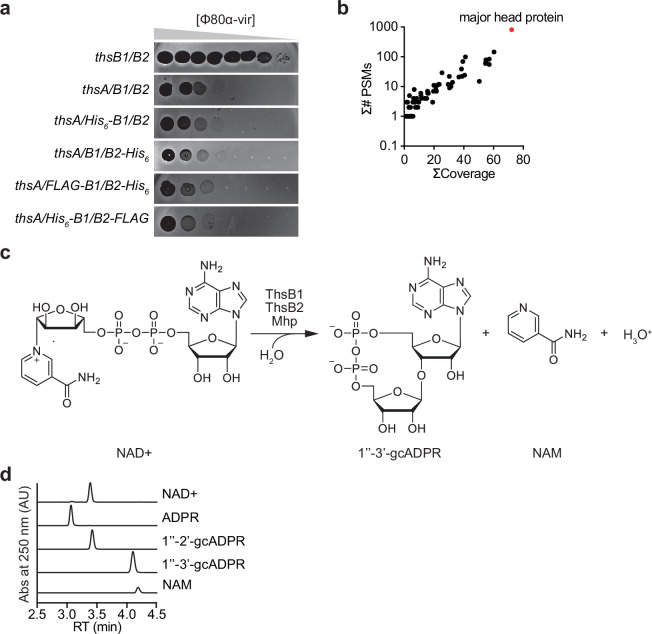
Validation of tagged constructs for defense activity, Mhp co-purification, NAD⁺ cyclization, and chemical standards. **(a)** Tenfold serial dilutions of Φ80a-vir phage on lawns of *S. aureus* RN4220 harboring plasmids carrying either untagged or hexahystidyl- or Flag-tagged versions of ThsB1 or ThsB2, along with untagged ThsA. **(b)** LC-MS/MS identification of ~35 kDa protein isolated with ThsB1 and ThsB2 from infected *S. aureus* RN4220 cells shown in Fig. 3b. Gel slices (n = 3) were subjected to reduction, alkylation, and in-gel digestion. Peptides were extracted before being injected for LC-MS/MS analysis. Search results against the staphylococcal phage ΦNM1 proteome are presented as the percentage of sequence coverage (ΣCoverage) versus the indication of unique identified peptides (ΣPSMs). Data for the phage Mhp is shown in red. **(c)** Cyclization reaction of oxidized nicotinamide adenine dinucleotide (NAD + ) mediated by the ThsB1/B2/Mhp complex, which yields 1”-3’- glyocyclic ADP-ribose (1”-3’-gcADPR) and nicotinamide (NAM). **(d)** HPLC analysis of different commercially available chemicals used in this study.

**Extended Data Fig. 4:**
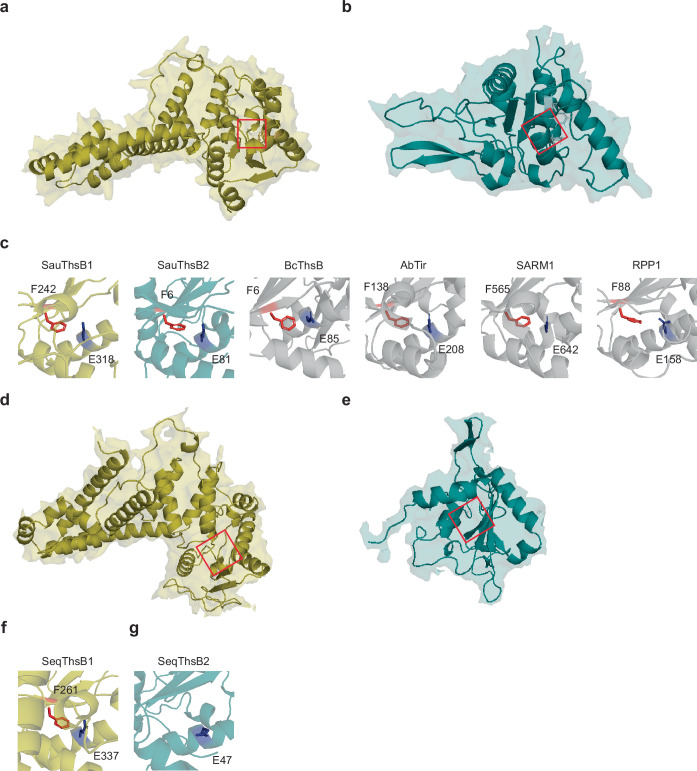
AlphaFold3-based structural analysis of ThsB1 and ThsB2. **(a)** Structure of ThsB1 generated by AlphaFold3 (pTM = 0.87), with the putative active site marked with a red square. **(b)** Same as **(a)** but for ThsB2 (pTM = 0.9). **(c)** Structural comparison of the predicted active sites of ThsB1 and ThsB2 (red squares in panels A and B, respectively) with the experimentally characterized active sites of different TIR proteins: *Bacillus cereus* ThsB (BcThsB; PDB: 6LHY), *Acinetobacter baumannii* TIR domain (AbTir; PDB: 7UXU), human sterile alpha and TIR motif containing preotein 1 (SARM1; PDB: 6O0R) and *Arabidopsis thaliana* resistance protein RPP1 (PDB: 7DFV). **(d)** Structure of Seq-ThsB1 (pTM = 0.85) generated by AlphaFold3, with the putative active site marked with a red square. **(e)** Same as **(d)** but for Seq-ThsB2 (pTM= 0.85). **(f-g)** Structures of the predicted active sites of Seq-ThsB1 and Seq-ThsB2 (red squares in panels **d** and **e**, respectively).

To determine whether Mhp directly interacts with the Sau–Thoeris sensors, we introduced hexahistidyl (His_6_) and 3× Flag tags to ThsB1 (N terminus) or ThsB2 (C terminus), which did not affect immunity (Extended Data Fig. 3a). We then expressed complementary tagged versions of ThsB1 and ThsB2 in a ΦNM1 lysogen. Western blot using an anti-Flag antibody of His_6_-tagged proteins obtained from lysates purified using a cobalt resin showed the formation of a stable complex between ThsB1 and ThsB2 only when the lytic cycle of ΦNM1 was induced, which depended on the presence of *gp43* (Fig. 3a). Importantly, sodium dodecyl–sulfate polyacrylamide gel electrophoresis (SDS–PAGE) of the pulled-down samples revealed the presence of a third protein that copurified with the ThsB1–ThsB2 complex (Fig. 3b), which was identified as Mhp via mass spectrometry (Extended Data Fig. 3b and Supplementary Data 2). In addition, the isolated complexes were incubated with NAD^+^ and found to catalyse the synthesis of 1″-3′-gcADPR (Extended Data Fig. 3c) only when the wild type prophage was induced—not in the absence of a prophage or during induction of ΦNM1(Δ*gp43*) (Fig. 3c,d and Extended Data Fig. 3d). Finally, alignment of AlphaFold structures for ThsB1 and ThsB2 (Extended Data Fig. 4a,b) to closely related, previously characterized TIR proteins (Extended Data Fig. 4c) identified a putative catalytic glutamate located across from a phenylalanine residue (E318/F242 in ThsB1 and E81/F6 in ThsB2; Extended Data Fig. 4c), which we mutated to glutamine or alanine, respectively. We found that both E81Q and F6A substitutions in ThsB2, but none of the ThsB1 mutations, disrupted immunity against Φ80α-vir in a plaquing assay (Fig. 3e). These results suggest that the catalytic activity of ThsB2 is required for the synthesis of the second messenger. Pull-down of ThsB2^F6A^-His_6_ from lysates of staphylococci infected with Φ80α-vir revealed that this mutation does not interfere with the formation of the Mhp–ThsB1–ThsB2 complex (Fig. 3f) but abrogates its cyclase activity in vitro (Fig. 3g). Altogether, these results demonstrate that ThsB1 and ThsB2 form a complex with Mhp during phage infection that stimulates ThsB2 cyclase activity to initiate the Thoeris immune response. By contrast, in uninfected hosts, ThsB1 and ThsB2 do not interact with each other.

### ThsB1 interacts with Mhp to recruit and activate ThsB2

**Fig. 4:**
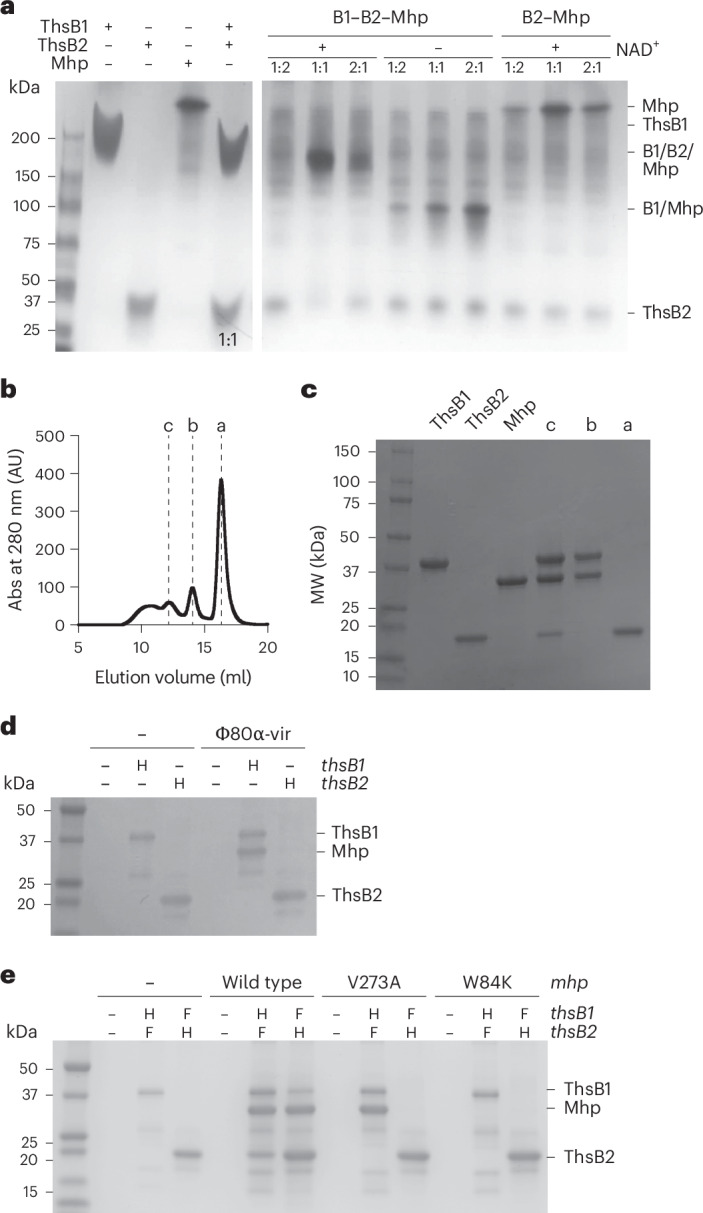
ThsB1 interacts with Mhp to recruit ThsB2. **a**, SDS–PAGE analysis of purified His_6_-ThsB1, ThsB2-His_6_ or Mhp, and His_6_-ThsB1 incubated with ThsB2-His_6_ (left). Native PAGE analysis of ThsB1, ThsB2 and Mhp with and without NAD^+^. Each lane is labelled with the molar ratio ThsB:Mhp (right). The strong bands that appear ~100 kDa in the presence of ThsB1–THsB2–Mhp and without NAD^+^ probably represent nonspecific aggregates or the ThsB1–Mhp complex that should form in the absence of NAD^+^. Free ThsB1 is expected but may not separate clearly from the other higher-order structures. **b**, SEC analysis of the Mhp–ThsB1–ThsB2 complex. Fractions corresponding to peaks marked as a,b and c were collected for SDS–PAGE analysis. **c**, SDS–PAGE analysis of purified His_6_-ThsB1, ThsB2-His_6_ or Mhp, and SEC fractions a, b and c. Molecular weight (MW) in kDa of protein markers are shown. **d**, Coomassie Blue-stained SDS–PAGE of proteins isolated from staphylococci expressing hexahystidyl- (H) tagged versions of either ThsB1 or ThsB2, uninfected or infected with Φ80α-vir, after cobalt resin affinity chromatography. Protein molecular weight (kDa) markers are shown. **e**, Coomassie Blue-stained SDS–PAGE of proteins isolated from staphylococci expressing hexahystidyl- (H) or 3× Flag- (F) tagged versions of ThsB1 and ThsB2 in the absence and presence of wild type, V273A or W84K Mhp expressed from a plasmid, after cobalt resin affinity chromatography.

**Extended Data Fig. 5:**
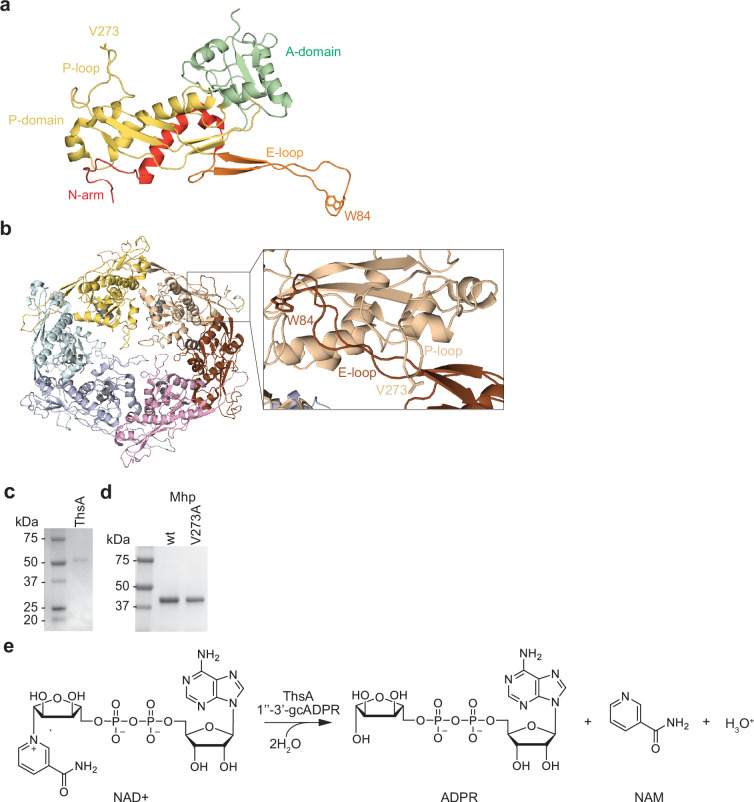
Characterization of V273A and W84K mutations. **(a)** Structure Φ80α Mhp monomer (PDB: 6B0X), colored according to the different structural domains (red, N-arm; yellow, P domain and loop; green, A-domain; orange, E-loop). **(b)** Structure of the hexameric form of Φ80α Mhp (PDB: 6B0X), with one capsomer interface highlighted. The position of the residues which mutations that led to immune evasion, V273 within the P-loop (tan) and W84 within the E-loop (brown), are shown (ipTM 0.47, pTM 0.52). **(c)** Coomassie Blue-stained SDS-PAGE of proteins purified from *S. aureus* expressing His_6_-ThsA (56.0 kDa) using a cobalt resin. Protein molecular weight (kDa) markers are shown. **(d)** Coomassie Blue-stained SDS-PAGE of Mhp proteins, wild type and V273A mutant, purified from staphylococci harboring pMhp plasmids, using PEG-enrichment. Protein molecular weight (kDa) markers are shown. **(e)** Hydrolysis reaction of oxidized nicotinamide adenine dinucleotide (NAD + ) mediated by ThsA when activated by 1”-3’-gcADPR, which yields ADP-ribose (ADPR) and nicotinamide (NAM).

To understand the formation and stoichiometry of the Mhp–ThsB1–ThsB2 complex, we purified the three proteins (Fig. 4a,c) to perform native polyacrylamide gel electrophoresis (PAGE). Mixtures of ThsB1/B2 and ThsB2/Mhp at different ratios failed to show the formation of a complex (Fig. 4a). Incubation of ThsB1, ThsB2 and Mhp, led to the generation of a stable complex only at the 1:1:1 ratio, only in the presence of NAD^+^, whereas free ThsB2 was detected at higher and lower Mhp concentrations. While a 1:1:2 ratio of ThsB1:ThsB2:Mhp should theoretically permit complex formation, we did not observe a distinct complex band under these conditions (Fig. 4a). This may reflect a dynamic equilibrium or instability of the complex in the presence of excess Mhp that could oligomerize and in turn cause the dissociation of the complex or the formation of alternate species not clearly resolved by native PAGE. The structure of Mhp from Φ80α (Mhp(Φ80α)) has previously been solved experimentally and demonstrated to be a hexamer^24^ (Extended Data Fig. 5a,b). We therefore performed size exclusion chromatography (SEC) and mass photometry to determine how many Mhp subunits were present in the complex assembled from individually purified subunits in the presence of NAD^+^. During chromatography, the sample separated into three main peaks (Fig. 4b), the contents of which were analysed using SDS–PAGE (Fig. 4c). Peak a contained only ThsB2 with a measured molecular mass of 47 ± 8.7 kDa, a value that suggests the presence of a dimer because ThsB2 mass is ~24.5 kDa. Peak b consisted of an Mhp–ThsB1 complex with a mass of 81 ± 20 kDa, which matches the expected molecular weight of a complex of monomers (~81.5 kDa). Peak c contained all three proteins with an estimated mass of 164 ± 88 kDa, which is difficult to assign to a particular complex stoichiometry given the high standard deviation (s.d.) of the measurement. The expected mass for a monomeric Mhp–ThsB1–ThsB2 complex is 106 kDa. These results suggest the formation of a more stable complex between Mhp and ThsB1 than ThsB2. We corroborated this observation in vivo through pull-down experiments using lysates of staphylococci harbouring a plasmid for the overexpression of Mhp (pMhp) in the presence of either His_6_-ThsB1 or ThsB2-His_6_, not both, which showed that the capsid protein copurifies with ThsB1 but not with ThsB2 (Fig. 4d).

We also tested the effect of Mhp mutations on complex formation. Pull-down of either His_6_-ThsB1 or ThsB2-His_6_, from lysates of cells also expressing 3× Flag-tagged versions of the other sensor as well as either wild type or the V273A mutant Mhp from the pMhp plasmid, showed that the escape mutation did not affect Mhp binding to ThsB1 but prevented the addition of ThsB2 to form the tri-partite complex (Fig. 4e). Residue V273 is located in the P-loop, located ~60 Å apart from the E-loop on the opposite side of the Mhp monomer^21,25^ (Extended Data Fig. 5a). To test for the importance of the E-loop in the assembly of the Thoeris sensor complex, we substituted W84 (Extended Data Fig. 5a,b) with lysine, a residue found in Mhp homologues at this position. When introduced in Φ80α-vir, the W84K mutation led to the formation of viable viral particles that also escaped Thoeris immunity (Extended Data Fig. 2a). In addition, introduction of pMhp^W84K^ in staphylococci harbouring the *ths* operon did not reduce NAD(H) levels (Fig. 2d), a result that demonstrates that the mutation prevents the activation of the Thoeris response. Finally, pull-downs of His_6_-ThsB1 or ThsB2-His_6_ after overexpression of Mhp^W84K^ showed that the mutant Mhp did not interact with either ThsB1 or ThsB2 (Fig. 4e).

Together, these results support a model in which (1) Mhp and ThsB1 form an initial subcomplex that requires an intact E-loop in Mhp, (2) ThsB2 is recruited by this subcomplex in a manner that depends on the P-loop of Mhp, and (3) ThsB2 cyclase activity is stimulated, possibly through interactions with ThsB1 that are mediated by the binding of NAD^+^. Given the 1:1 stoichiometry of the Mhp–ThsB1 subcomplex, we propose that this stepwise model of Thoeris activation occurs in the context of an Mhp monomer; however, whether any of these steps can also involve interactions with Mhp hexamers is not known.

### Mhp and ThsB1 are sufficient to activate ThsB2 in vitro

**Fig. 5:**
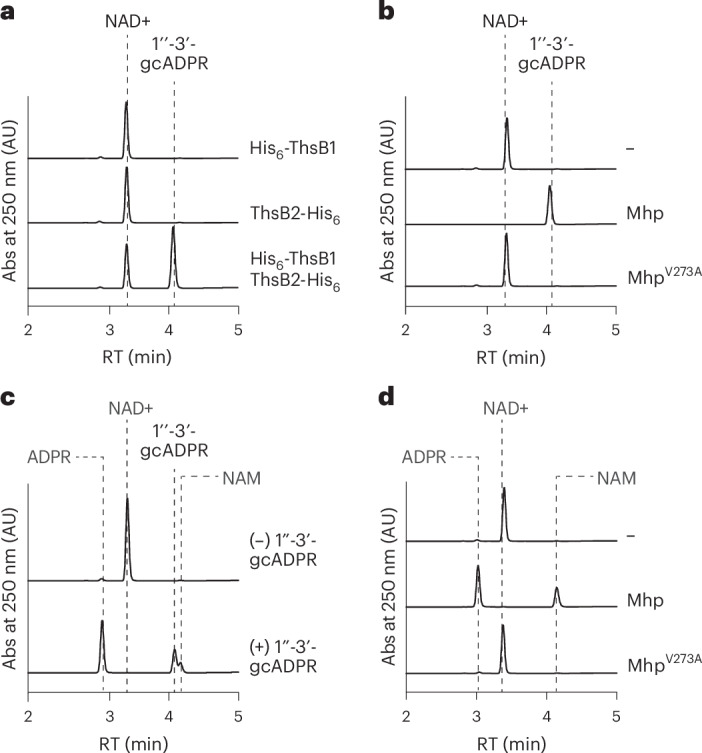
Mhp and ThsB1 are sufficient to stimulate ThsB2 cyclase activity in vitro*.* **a**, HPLC analysis of the products resulting from the incubation of purified His_6_-ThsB1, ThsB2-His_6_ or both with NAD^+^ and Mhp. RT of reactants and products is marked with dashed lines. **b**, The same as in **a** but after incubation of both His_6_-ThsB1 and ThsB2-His_6_, alone (−) or in the presence of purified wild type or V273A mutant Mhp. **c**, HPLC analysis of the products resulting from the incubation of purified His_6_-ThsA with (+) or without (−) commercially available 1″-3′-gcADPR. RT of reactants and products is marked with dashed lines. **d**, HPLC analysis of the products resulting from the incubation of purified His_6_-ThsA with the products of the reactions shown in **b**, obtained after mixing ThsB2-His_6_, ThsB2^F6A^-His_6_, NAD^+^ and wild type or V273A mutant Mhp. RT of reactants and products is marked with dashed lines. ADPR, adenosine diphosphate ribose; NAM, nicotinamide.

We investigated the minimal requirements for the Sau–Thoeris response by performing biochemical reactions with individually purified proteins His_6_-ThsB1, ThsB2-His_6_, His_6_-ThsA (Extended Data Fig. 5c), Mhp and Mhp^V273A^ (Extended Data Fig. 5d). High-performance liquid chromatography (HPLC) analysis of the reaction products showed that wild type Mhp, but not Mhp^V273A^, stimulates the production of 1″-3′-gcADPR by ThsB2 only in the presence of ThsB1 (Fig. 5a,b). His_6_-ThsA cleaves NAD^+^ into ADPR and NAM^5,8^ (Extended Data Fig. 5e) both in the presence of commercial (Fig. 5c) and in vitro-synthesized (by the ThsB sensors and Mhp, Fig. 5d) 1″-3′-gcADPR. These data demonstrate that Mhp alone, without any other cellular component, is required to stimulate the synthesis of 1″-3′-gcADPR by ThsB1 and ThsB2, which in turn activates ThsA to convert NAD^+^ into ADPR and NAM.

### Sau–Thoeris is activated by Mhp of diverse staphylococcal phages

**Fig. 6:**
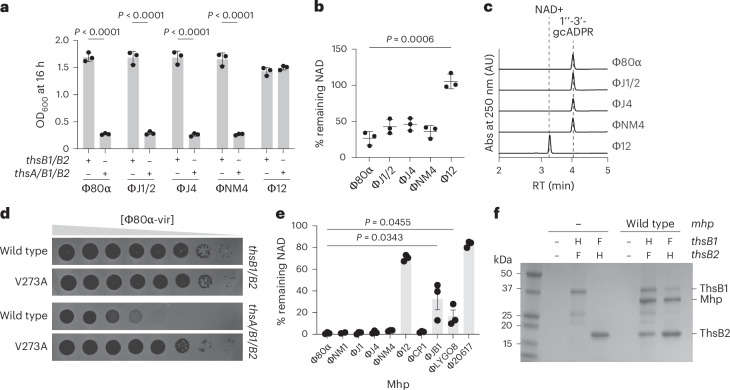
ThsB1 and ThsB2 recognize diverse capsid proteins. **a**, Growth of *S. aureus* RN4220 harbouring plasmids carrying either an incomplete (*thsB1/B2*) or full (*thsA/B1/B2*) Thoeris operon in the presence of a second plasmid expressing Mhp from different staphylococcal phages, determined as the OD_600_ of the cultures 16 h after addition of IPTG. Mean ± s.d. of three biological replicates is reported; *P* values (*P* < 0.0001) were obtained using an unpaired, two-tailed, *t*-test comparing groups with partial and full Thoeris. **b**, Measure of percentage remaining NAD(H), calculated as the ratio of the concentration of NAD^+^ and NADH detected in staphylococci harbouring a plasmid carrying a full (*thsA/B1/B2*) Thoeris operon to the value detected in the presence of an incomplete (*thsB1/B2*) system, after induction of a second plasmid expressing Mhp from different staphylococcal phages. Mean ± s.d. of three biological replicates is reported; *P* value was obtained using an unpaired, two-tailed, *t*-test. **c**, HPLC analysis of the products resulting from the incubation of purified ThsB2-His_6_ and ThsB2^F^_6_^A^-His_6_ with NAD^+^, in the presence of purified Mhp from different staphylococcal phages. RT of reactants and products is marked with dashed lines. **d**, Tenfold serial dilutions of Φ80α-vir or Mhp^V273A^ escaper phage on lawns of *S. aureus* RN4220 carrying either an incomplete (*thsB1/B2*) or full (*thsA/B1/B2*) Thoeris operon from *S. equinus* on a pE194-based plasmid under the control of an IPTG-inducible promoter. **e**, Measure of percentage remaining NAD(H), calculated as the ratio of the concentration of NAD^+^ and NADH detected in staphylococci harbouring one plasmid carrying *thsA/B1/B2* from *S. equinus* and another carrying an *mhp* from various *Staphylococcus* or *Streptococcus* phages compared with the value detected in the absence of any of the *ths* genes, after induction of Mhp expression with IPTG. Mean ± s.d. of three biological replicates is reported; *P* value was obtained using an unpaired, two-tailed, *t*-test. **f**, Coomassie Blue-stained SDS–PAGE of proteins isolated from staphylococci expressing Seq–ThsB2-Flag and Seq–ThsB1-His_6_ with or without overexpression of Mhp from Φ80α-vir, after cobalt resin affinity chromatography. Protein molecular weight (kDa) markers are shown.

**Extended Data Fig. 6:**
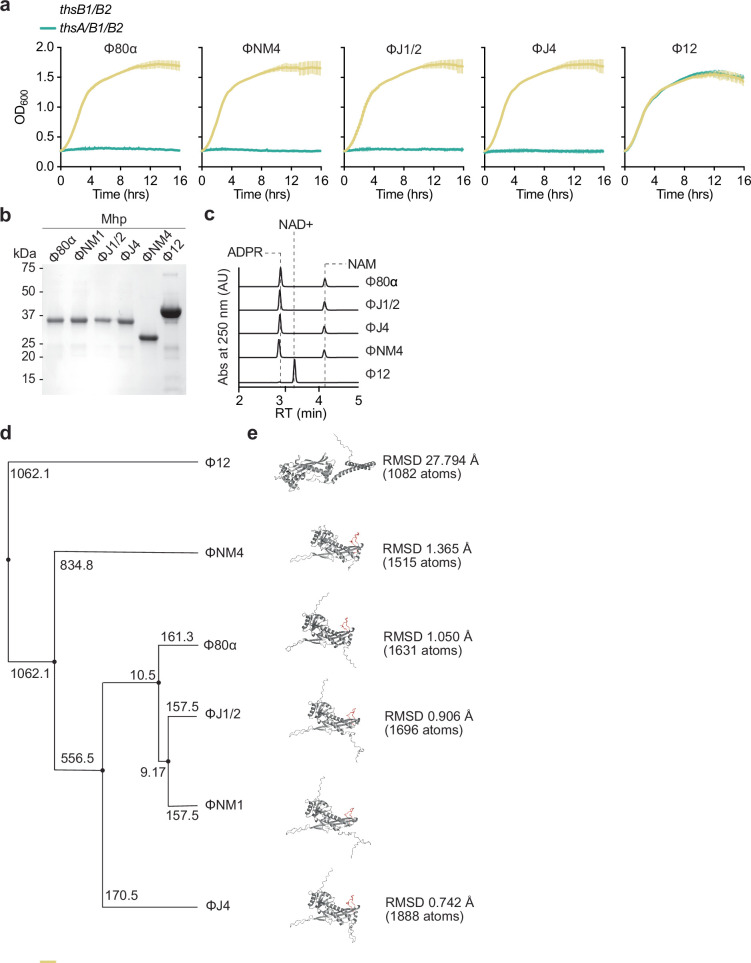
Mhp from diverse staphylococcal phages are sensed by Sau–Thoeris. **(a)** Growth of *S. aureus* RN4220 harboring plasmids carrying either an incomplete (*thsB1/B2*) or full (*thsA/B1/B2*) Thoeris operon in the presence of a second plasmid expressing Mhp from different staphylococcal phages, determined as the OD_600_ of the cultures after addition of IPTG. The values at the end of the curves, at 16 hours, were used to make the bar graphs shown in Fig. 6a. Mean of +/- S.D. of three biological replicates is reported. **(b)** Coomassie Blue-stained SDS-PAGE of Mhp proteins, purified from staphylococci harboring pMhp plasmids using PEG-enrichment. Protein molecular weight (kDa) markers are shown. **(c)** HPLC analysis of the products resulting from the incubation of purified His_6_-ThsA with the products of the reactions shown in Fig. 6c, obtained after mixing ThsB2-His_6_, ThsB2^F6A^-His_6_, NAD^+^ and purified Mhp from different staphylococcal phages. Retention times (RT) of reactants and products are marked by dotted lines. **(d)** Phylogenetic tree of the staphylococcal Mhps used in this study, generated using EMBL-EBI Muscle multiple sequence alignment tool. The branch numbers indicate genetic distance from ΦNM1 Mhp calculated by the software. **(e)** Alphafold3 prediction of the monomeric form of each Mhp encoded by the different phages. Structural distance is shown as the RMSD of the different Mhps compared to ΦNM1 Mhp (pTMs: Φ12γ3 pTM = 0.76, ΦNM4 pTM = 0.80, Φ80α pTM = 0.79, ΦJ1 pTM = 0.78, ΦNM1 pTM = .75, ΦJ4 pTM = 0.81).

Sau–Thoeris provides immunity against Φ80α-vir, ΦNM1γ6, ΦNM4γ4, ΦJ1, ΦJ2 and ΦJ4 but not Φ12γ3 (Fig. 1b). We cloned the gene encoding Mhp of all these phages (ΦJ1 and ΦJ2 Mhp are identical) and found that, with the exception of Mhp(Φ12γ3), Mhp overexpression was sufficient to cause growth arrest of staphylococcal cultures harbouring the full *thsA/B1/B2* operon (Fig. 6a and Extended Data Fig. 6a), as well as NAD(H) depletion (Fig. 6b). We also purified Mhp from staphylococci infected with these phages (Extended Data Fig. 6b) and found that all but Mhp(Φ12γ3) stimulated purified His_6_-ThsB1 and ThsB2-His_6_ to convert NAD^+^ to 1″-3′-gcADPR in vitro (Fig. 6c). In turn, the cyclic nucleotide produced was able to induce the cleavage of NAD^+^ by His_6_-ThsA (Extended Data Fig. 6c). A phylogenetic tree of the six Mhps, using Mhp(ΦNM1γ6) as reference, demonstrated that Mhp(Φ12γ3) is the most distant member of this group of structural proteins (Extended Data Fig. 6d). Likewise, AlphaFold 3 (ref. ^26^) structural predictions showed that while Mhps from phages Φ80α-vir, ΦNM4γ4, ΦJ1/2, ΦJ4 and ΦNM1γ6 display very similar folding, with root mean square deviation (RMSD) values ~1 Å, there was a marked structural variation for Mhp(Φ12γ3), with an RMSD over 27 Å (Extended Data Fig. 6e). Therefore, we believe that the sequence and structural divergence of Mhp(Φ12γ3) prevents its recognition by the Sau–ThsB sensors. However, our results demonstrate that diverse staphylococcal phages with structurally conserved capsid proteins are recognized by Sau–Thoeris to trigger defense.

### *Streptococcus equinis* Thoeris immunity is activated by Mhp

**Extended Data Fig. 7:**
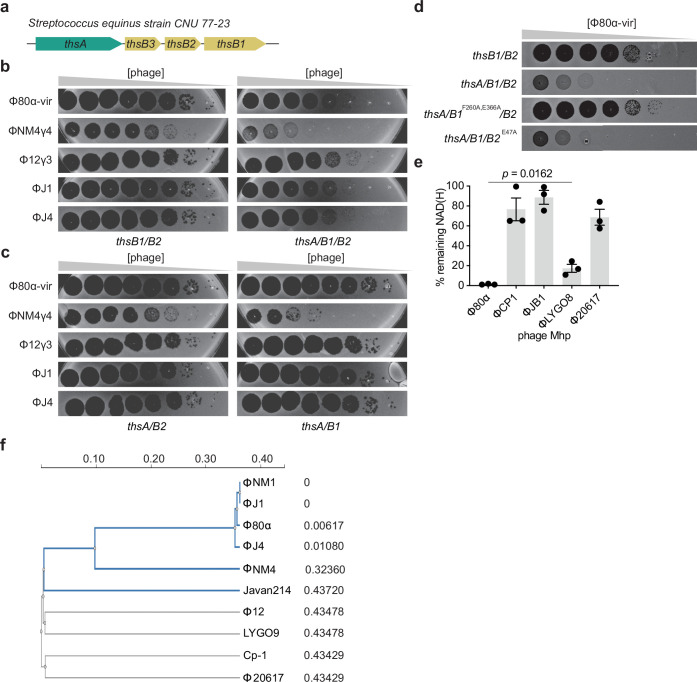
The *Streptococcus equinis* Thoeris system is also activated by Mhp. **(a)** Schematic of the Thoeris operon present in the *Streptococcus equinus* strain CNU 77-23. It contains homologs of ThsA, ThsB1, and ThsB2, and an additional TIR-containing protein labelled ThsB3. **(b)** Tenfold serial dilutions of staphylococcal phages on lawns of *S. aureus* RN4220 carrying either incomplete (*thsB1/B2*) or full (*thsA/B1/B2*) Thoeris operon from *S. equinus* on a pE194-based plasmid under the control of an IPTG-inducible promoter. **(c)** Same as **(**b**)** with lawns od cells carrying *thsA/B1* or *thsA/B2*. **(d)** Same as **(b)** with lawns of *S. aureus* RN4220 carrying either an incomplete (*thsB1/B2*) or full (*thsA/B1/B2*) Thoeris operon from *S. equinus*, compared to the full system with catalytic mutants (*thsA/B1*^*F260A,E366A*^*/B*2), (*thsA/B1/B2*^*E47A*^*)*. **(e)** Measure of percentage remaining NAD(H), calculated as the ratio of the concentration of NAD+ and NADH detected in staphylococci harboring one plasmid carrying a full *ths operon* from *S. aureus* and another carrying an *mhp* from various *Staphylococcus* or *Streptococcus* phages compared to the value detected in the absence of any of the *ths* genes, after induction of Mhp expression with IPTG. Mean of +/- S.D. of three biological replicates is reported; *p* value was obtained using an unpaired, two-tailed, *t*-test. **(f)** Phylogenetic tree of the Mhps from both staphylococcal and streptococcal phages used in this study, generated using EMBL-EBI Muscle multiple sequence alignment tool. The branch numbers indicate genetic distance from ΦNM1 Mhp calculated by the software.

To test if our results apply to other Thoeris defense systems, we investigated a *ths* operon present in *Streptococcus equinis* CNU77-23 (Seq–Thoeris, Extended Data Fig. 7a) encoding ThsB1 and ThsB2 proteins with significant sequence homology to their staphylococcal counterparts (48% and 35% of sequence identity, respectively), as well as similar predicted structures (Extended Data Fig. 4d,e). The operon contains a third protein of unknown function, referred to here as ThsB3. We transformed staphylococci with plasmids carrying different *ths* gene combinations (excluding *thsB3*) and performed plaque assays. We found that Seq–Thoeris provided strong protection against ΦNM4γ4 (approximately three orders of magnitude of plaque-forming unit (PFU) reduction), and weaker immunity against Φ80α-vir, ΦJ1 and ΦJ4 (approximately one to two orders of magnitude of PFU reduction as well as smaller plaques), but no defense against Φ12γ3 (Extended Data Fig. 7b). Interestingly, ThsB1 was sufficient for defense against ΦNM4γ4 (Extended Data Fig. 7c), a result that suggested that Seq–ThsB1, as opposed to Sau–ThsB1 (Fig. 4a), is responsible for the synthesis of the second messenger. Using AlphaFold 3 to obtain structural models of Seq–ThsB1 and Seq–ThsB2 (Extended Data Fig. 4d,e) we determined the putative residues required for catalysis: F260 and E336 in Seq–ThsB1 (Extended Data Fig. 4f) and only E47 in Seq–ThsB2 (Extended Data Fig. 4e,g). Mutations that generated alanine substitutions of these residues demonstrated that, as we suspected, the ability of Seq–Thoeris to reduce plaque formation depended on the catalytic activity of ThsB1 but not ThsB2 (Extended Data Fig. 7d).

Plaque assays with Φ80α-vir(*mhp*^V273A^) revealed that this mutant phage can also evade Seq–Thoeris immunity (Fig. 6d), a result that involves Mhp in the activation of this system as well. Indeed, expression of Mhp from Φ80α-vir, ΦNM1γ6, ΦNM4γ4, ΦJ1/2 and ΦJ4, but not Φ12γ3, promoted NAD^+^ depletion in staphylococci harbouring the Seq–Thoeris system (Fig. 6e). Moreover, expression and pull-down of His_6_-tagged versions of Seq–ThsB1 or Seq–ThsB2 from cytoplasmic extracts obtained in the presence or absence of Mhp(Φ80α) overexpression demonstrated the formation of a complex between ThsB1, ThsB2 and Mhp (35 kDa) (Fig. 6f). Finally, we expressed Mhps from diverse streptococcal phages in staphylococci harbouring Seq–Thoeris and found that the capsid proteins from *Streptococcus pneumoniae* phage Cp-1, *S. equinis* phage Javan214 and *Streptococcus agalactiae* prophage LYG09, but not Mhp from *Streptococcus thermophilus* phage Φ20617, induced NAD(H) depletion (Fig. 6e). Interestingly, when we tested the streptococcal Mhps in the presence of the Sau–Thoeris system (Extended Data Fig. 7e), only phage Javan214 Mhp, phylogenetically the closest to the staphylococcal Mhp, was able to mediate a reduction of NAD(H) levels (Extended Data Fig. 7f). Altogether, these results demonstrate that different TIR-domain cyclases have evolved to recognize phage capsid proteins to initiate the Thoeris immune response.

## Discussion

Here, we investigated how the *S. aureus* Thoeris system is activated by phages during infection. We found that the Mhps of different staphylococcal *Siphoviridae* phages that are susceptible to Thoeris defense mediate the association of the TIR-containing proteins ThsB1 and ThsB2. Our experimental data support a model in which binding of ThsB1 to the Mhp leads to the recruitment of ThsB2 and stimulation of its cyclase activity, which converts NAD^+^ into the second messenger 1″-3′-gcADPR. The results that validate this mechanism of Sau–Thoeris activation are: (1) ThsB1 and ThsB2 do not interact with each other in the absence of Mhp (Fig. 3a), (2) Mhp expression leads to its association with both ThsB1 and ThsB2 (Fig. 3b), (3) in vivo, in the absence of ThsB2, ThsB1 interacts with Mhp, but ThsB2 does not interact with Mhp in the absence of ThsB1 (Fig. 4a), (4) in vitro, purified Mhp and ThsB1 form a stable subcomplex with a 1:1 stoichiometry (Fig. 5c) and (5) the ThsB2 putative cyclase active site is critical for the synthesis of gcADPR (Fig. 4a). Several features of this model will require further investigation (Supplementary Discussion).

Immunological logic dictates that bacterial defense systems sense conserved molecules produced during phage infection^27,28^. Conservation of immunological targets ensures (1) that the activating molecules are present in many viruses, making the immune system useful against a broad range of phages, and (2) that the target is essential for optimal phage propagation and therefore difficult to mutate, reducing the chances of viral escape. We believe that the targeting of Mhp by Sau–Thoeris meets both evolutionary requirements. Mhp from Φ80α is highly conserved among many staphylococcal phages (Extended Data Fig. 6d), and four out of five homologues were able to activate Sau–Thoeris (Fig. 6 and Extended Data Fig. 6). Mhp is also essential for Φ80α propagation (Extended Data Fig. 2d), and we were able to find only two escape mutations, V273A and W84K, using either sequential infections of staphylococci carrying the Sau–Thoeris system or genetic engineering based on sequence conservation, respectively. Mhp mutations that escape Sau–Thoeris cannot disrupt capsid formation but probably result in the generation of an altered conformation of the viral procapsids. Although such mutations are infrequent, they can accumulate as a consequence of the evolutionary arms race between phages and their prokaryotic hosts, and most probably will result in changes in capsid morphology. The *S. aureus* phages in this study belong to the *Siphoviridae* group, which can be classified into distinct serogroups on the basis of morphological differences in their head structures^29^. Notably, phages Φ80α, ΦNM1, ΦNM4, ΦJ1, ΦJ2 and ΦJ4, which activate Sau–Thoeris, belong to serogroup B and have isometric capsids. By contrast, Φ12, of which the Mhp avoids triggering TIR-mediated defense, belongs to serogroup A and has a more prolate head structure, which is lengthened in one direction^29^. We believe that the evasion of Sau–Thoeris immunity, and possibly other defense systems that are activated by capsid proteins, represents one important evolutionary force in the differentiation of the Φ12 capsid structure and, more generally, in the generation of structural diversity in staphylococcal phages. Our finding that Seq–Thoeris also senses Mhps from streptococcal phages suggests that similar considerations apply for the impact of the evolutionary arms race between bacteria and their phages on the structural diversity of viral capsids.

## Methods

### Bacterial strains and growth conditions

The bacterial strains used in this study are listed in Supplementary Data 3. *S. aureus* strain RN4220 (ref. ^29^) was grown at 37 °C with shaking (220 revolutions per minute) in Brain Heart Infusion (BHI) broth, supplemented with chloramphenicol (10 mg ml^−1^) or erythromycin (10 mg ml^−1^) to maintain pC194-based^23^ or pE194-based plasmids^12^, respectively. Cultures were supplemented with erythromycin (5 mg ml^−1^) to select for strains with chromosomally integrated Sau–Thoeris or Sau–*thsB1/B2*. Gene expression was induced by the addition of 1 mM IPTG, where appropriate.

### Bacteriophage propagation

The bacteriophages used in this study are provided in Supplementary Data 3. To generate a high-titre phage stock, an overnight culture of *S. aureus* RN4220 was diluted 1:100 and outgrown to mid-log phase (~90 min) in BHI broth supplemented with 5 mM CaCl_2_. The culture was diluted to an optical density measurement at 600 nm (OD_600_) of 0.5 (~1 × 10^8^ CFU ml^−1^). The culture was infected by adding phage at an MOI of 0.1 (~1 × 10^7^ PFU ml^−1^) or by inoculating with either a single picked plaque or scrape of a frozen stock. The infected culture was grown at 37 °C with shaking and monitored for lysis (full loss of turbidity was typically observed after ~3-4 h). Culture lysates were centrifugated (4,300*g* for 10 min) to pellet cellular debris. The supernatant was collected, passed through a sterile membrane filter (0.45 mm) and stored at 4 °C. Phage concentrations were determined by serially diluting the obtained stock in tenfold increments and spotting 2.5 ml of each dilution on BHI soft agar mixed with RN4220 and supplemented with 5 mM CaCl_2_. After incubation overnight at 37 °C, individual plaques (that is, zones of no bacterial growth) were counted, and the viral titre was calculated.

### Molecular cloning

The plasmids (and details of their construction) and the oligonucleotide primers used in this study are provided in Supplementary Data 3. The coding sequences of Sau–Thoeris and phage gene products were obtained from G blocks, genomic DNA preparations or phage stocks, respectively.

### Chromosomal integration of Sau–Thoeris

Sau–*thsA/B1/B2* or Sau–*thsB1/B2*, along with an erythromycin resistance (ermR) cassette, was integrated into the *hsdR* gene (which encodes the defective R-subunit of the restriction–modification system in *S. aureus* RN4220), an insertion site which was previously shown to not impact growth^30^. *Sau–thsA/B1/B2-ermR* and *Sau–thsB1/B2-ermR* were amplified from the plasmids pDVB223 and pCF11, respectively, using primers oCR482 and oCR483 or oCR484, which were flanked with loxP sites at both ends followed by 60-bp homology regions to *hsdR*. Electrocompetent *S. aureus* RN4220 cells harbouring the recombineering plasmid pPM300 (ref. ^14^) were electroporated with 1–2 mg of polymerase chain reaction (PCR) product and selected for with erythromycin (5 mg ml^−1^). Potential integrants were screened using colony PCR as well as for functional immunity and then verified by Sanger sequencing.

### Prophage recombineering

The prophage strains and the oligonucleotide primers used in this study are provided in Supplementary Data 3. A chloramphenicol resistance cassette flanked by loxP sites and 60 bp homology regions were integrated within codons for phage genes of interest corresponding to the homology overhangs. The loxP chloramphenicol resistance was amplified from a G-block (Azenta), using primers oCR24 and oCR25, oCR26 and oCR37, oCR30 and oCR31, oCR32 and oCR33, oCR485 and oCR486, oCR487 and oCR488, oCR489 and oCR490, oCR491 and oCR492, oCR493 and oCR494, and oCR495 and oCR496, which were all flanked with 60-bp homology regions. Electrocompetent *S. aureus* RN4220 cells harbouring the recombineering plasmid pPM300 were electroporated with 1–2 mg of PCR product and selected for with chloramphenicol (5 mg ml^−1^). Potential integrants were screened using colony PCR as well as for functional immunity, and then verified by Sanger sequencing.

### Soft-agar phage infection

A total of 100 ml of an overnight bacterial culture was mixed with 5 ml BHI soft agar supplemented with 5 mM CaCl_2_ and poured onto BHI agar plates to solidify at room temperature (~15 min). Phage lysates were serially diluted tenfold and 2.5 ml was spotted onto the soft agar surface. Once dry, plates were incubated at 37 °C overnight and visualized the next day. Individual plaques (zones of no bacterial growth) were enumerated manually.

### Liquid culture phage infection

Overnight cultures were diluted 1:100 in BHI supplemented with 5 mM CaCl_2_ and the appropriate antibiotic for selection, outgrown at 37 °C with shaking to mid-log phase (~90 min) and normalized to an OD_600_ of 0.5. For the desired MOI, a calculated volume of phage stock was added to each culture and 150 ml was seeded into each well of a 96-well plate. OD_600_ was measured every 10 min in a microplate reader (TECAN Infinite 200 PRO) at 37 °C with shaking.

### Generation of Φ80a-vir(*mhp*^*W84K*^)

Wild type Φ80α-vir was passaged on a liquid culture of *S. aureus* RN4220 harbouring a plasmid (pCR186) encoding the *mhp*^*W84K*^ gene flanked by 500-nt upstream and downstream homology arms corresponding to Φ80α *gp46* and *gp48*, respectively. To isolate individual plaques, the lysed culture supernatant was spotted onto a lawn of RN4220 harbouring a type II-A Sau CRISPR-Cas targeting plasmid (pCR187) in BHI soft agar for counterselection against wild type phage and enrichment of Φ80α-vir::*mhp*^*W84K*^. The mutation was confirmed by Sanger sequencing.

### Protein expression and purification

ThsA, ThsB1 and ThsB2 were expressed and purified using the following approach: transformed *S. aureus* RN4220 was grown in BHI broth with 1 mM IPTG at 37 °C with shaking to an OD_600_ of 1.0, at which point the culture was cooled on ice for 10 min and bacteria were collected. Pellets were resuspended in lysis buffer (25 mM Tris pH 7.4, 100 mM NaCl, 10% glycerol, 2 mM β-mercaptoethanol and 5 mM MgSO_4_) and subjected to a single freeze–thaw cycle. The cells were incubated at 37 °C with lysostaphin, DNase I, and EDTA-free protease inhibitor cocktail (Millipore, stock keeping unit no. 11873580001) for 30 min. After incubating, the cells were lysed using sonication (70% amplitude, 10 s on/off, 2 min total). Lysates were clarified by centrifugation and applied to cobalt affinity resin. After binding, the resin was washed extensively with high salt lysis buffer (500 mM) before elution with lysis buffer containing 200 mM imidazole. Eluted proteins were subjected to overnight 4 °C dialysis into reaction buffer (25 mM Tris pH 7.4, 100 mM NaCl, 10% glycerol and 2 mM β-mercaptoethanol). The next day, proteins were concentrated using 10,000 MWCO centrifugal filters (Amicon). Purified proteins were visualized by SDS–PAGE and used for downstream in vitro assays.

### Purification of native Mhps

Native Mhps from Φ80α, ΦNM1, ΦNM4, ΦJ1, ΦJ2, ΦJ4 and Φ12 were expressed and purified either according to an established protocol^21,25^ using *S. aureus* cells harbouring pMhp or as follows: polyethylene glycol-precipitated phage (PEG) particles (10% (wt/vol) PEG8000 overnight at 4 °C, light centrifugation to collect precipitated particles the next day) were resuspended in unfolding buffer (4 M guanidine-HCl, 50 mM Tris-HCl pH 8.0 and 150 mM NaCl). Proteins were then layered on a 10–40% sucrose gradient and separated by centrifugation at 100,000*g* for 2 h. The gradients were manually fractionated from the top, and each fraction was analysed using SDS–PAGE. Fractions with capsid protein were subjected to dialysis into refolding buffer (25 mM Tris pH 7.4, 100 mM NaCl, 10% glycerol, 2 mM β-mercaptoethanol and 5 mM MgSO_4_) overnight at 4 °C. The final precipitate was removed by centrifugation, and the supernatant was used for downstream enzymatic assays.

### Nucleotide synthesis assays

Nucleotide synthesis assays were performed using a variation of the method described in ref. ^9^. The final reactions (25 mM Tris pH 7.4, 100 mM NaCl, 10% glycerol, 2 mM β-mercaptoethanol, 100 µM NAD^+^, 1 µM head protein and 1 μM enzyme) were started with the addition of enzyme. All reactions were incubated for 2 h at 37 °C. To isolate the gcADPR product for HPLC analysis, nucleotide synthesis reaction conditions were scaled up to 200 µl reactions. Reactions were incubated with gentle shaking for 2 h at 37 °C. Following incubation, reactions were filtered through a 3,000 MWCO centrifugal filter (Amicon) to remove protein and immediately used for HPLC analysis.

### ThsA NADase assay

NADase assays were performed with a 1:20 dilution of crude ThsB product or 100 nM purified 1″-3′-gcADPR (Biolog) diluted into final reactions (25 mM Tris pH 7.4, 100 mM NaCl, 10% glycerol, 2 mM β-mercaptoethanol, 100 μM NAD^+^ and 1 μM enzyme) and were started with the addition of enzyme. All reactions were incubated for 2 h at 37 °C. To isolate the degradation products for HPLC analysis, nucleotide synthesis reaction conditions were scaled up to 200 µl reactions. Reactions were incubated with gentle shaking for 2 h at 37 °C. Following incubation, reactions were filtered through a 3,000 MWCO centrifugal filter (Amicon) to remove protein and immediately used for HPLC analysis.

### NAD^+^ colorimetric assay

Detection of NAD from cell lysates was performed using an NAD^+^/NADH colorimetric assay kit (Abcam, ab65348). To generate lysates for analysis, an overnight culture of *S. aureus* RN4220 with partial or full Thoeris was diluted 1:100 and outgrown to mid-log phase (~90 min) in BHI broth supplemented with 5 mM CaCl_2_. The culture was diluted to an OD_600_ of 0.3. The culture was either infected by adding phage at MOI 1, or a prophage was induced with the addition of 1 μg ml^−1^ mitomycin C (MMC). The infected or induced cultures were grown at 37 °C with shaking for 1–2 h. Cell count was normalized to the lowest value after OD_600_ measurement. Pelleted cells were resuspended in 1× phosphate-buffered saline with lysostaphin. After incubating cells at 37 °C for 45 min, the resulting lysate was used for analysis and processed according to the manufacturers protocol.

### Nucleotide HPLC analysis

Reaction products were analysed using the 1460 HPLC system (Agilent) with a diode array detector at 260 nm. Sample (10 μl) was loaded onto a C18 column (100 × 2.0 mm, S-3 μm, 12 nm; YMC) equilibrated in 60 mM KH_2_PO_4,_ 40 mM K_2_HPO_4_ buffer. Separation was performed at a flow rate of 1.2 ml min^−1^ using a gradient programme for mobile phase (acetonitrile): 0–10 min.

### Structural prediction and analysis

The amino acid sequences of Thoeris proteins or Φ80α-vir, ΦNM1, ΦNM4, ΦJ1, ΦJ2, ΦJ4, Φ12, Cp-1, Javan214, Φ20617 and LYG09 Mhps were used to seed a Position-Specific Iterative Basic Local Alignment Search Tool search of the National Center for Biotechnology Information nonredundant protein and conserved domain databases (composition-based adjustment, E-value threshold 0.01). A structure for all Mhps was predicted using AlphaFold 3 as a hexamer. While the ipTM and pTM values were between 0.2 and 0.6 rather than above 0.8, which would represent a high confidence prediction, a structural alignment of the AlphaFold 3-predicted Mhp hexamer and the solved structure of the Φ80α Mhp hexamer (Protein Data Bank identifier 6B0X) gave RMSD values <1 Å for the monomer and <2 Å for the hexamer. Following structure determination, pairwise structural comparison of the rank 0 models was performed using PyMol. All predicted structures were compared to the solved structure of the Φ80α prohead (Protein Data Bank identifier 6B0X). The ConSurf database was used to visualize and pinpoint conserved structural and functional features of the major heads.

### SEC analysis and mass photometry

For oligomeric state characterization via size exclusion chromatography coupled with mass photometry, 500 μl purified protein or complex (1–2 mg ml^−1^) was injected onto a Superdex 200 Increase 10/300 GL column (GE Healthcare, Cytiva) using a buffer composed of 25 mM Tris (pH 7.5), 200 mM NaCl, 5% glycerol, and 1 mM dithiothreitol. Immediately before mass photometry, protein stocks were diluted in the same buffer (unless otherwise specified), resulting in typical working concentrations of 100–200 nM, adjusted on the basis of the dissociation properties of each assembly. Each sample was analysed using a new flow chamber to prevent cross-contamination. To establish focus, fresh buffer was first flowed into the chamber; the focal point was identified and then maintained via an autofocus system using total internal reflection throughout the measurement. For each acquisition, 15 μl of the diluted protein sample was introduced into the flow chamber; following autofocus stabilization, movies of either 60 or 90 s were recorded. Each sample was independently measured at least three times (*n* ≥ 3). All measurements were conducted on comparable mass photometry instruments, with most data acquired using a OneMP mass photometer (Refeyn, Oxford, UK).

### Time-lapse fluorescence microscopy

*S. aureus* cells harbouring incomplete (*thsB1/B2*) or full (*thsA/B1/B2*) Sau–Thoeris were loaded onto microfluidic chambers using the CellASIC ONIX2 microfluidic system. After cells became trapped in the chamber, they were supplied with BHI medium with 5 mM CaCl_2_ under a constant flow of 5 ml hr^−1^. After 1 h, GFP-tagged Φ80α-vir was flowed through the chambers for 1 h, before switching back to growth medium. Phase contrast images were captured at 1,000x magnification every 2 min using a Nikon Ti2e inverted microscope equipped with a Hamamatsu Orca-Fusion SCMOS camera, and the temperature-controlled enclosure was set to 37 °C. GFP was imaged using a GFP filter set using an Excelitas Xylis light emitting diode Illuminator set to 2% power, with an exposure time of 100 ms. Images were aligned and processed using the NIS Elements software.

### Generation and isolation of escaper bacteriophages

Overnight cultures of *S. aureus* RN4220 were diluted 1:100, outgrown at 37 °C with shaking for 1 h, and infected with Φ80α-vir (MOI 1) for 20 min. Cultures were allowed to lyse for 3 h before pelleting debris and sterile-filtering the supernatant to obtain phage. A total of 100 ml RN4220 overnight cultures harbouring Sau–Thoeris were infected with a high-titre mutant phage library in BHI soft agar and then plated. All plaques were collected, and the soft-agar infection was repeated five times. After the fifth passage at 37 °C overnight, individual phage plaques were picked from the top agar and resuspended in 50 ml BHI liquid medium. Phage lysates were further purified over two rounds of passaging on RN4220 harbouring Sau–Thoeris. Genomic DNA from high-titre phage stocks was extracted using previously described methods^31^ and was submitted to SeqCenter for whole genome sequencing and assembly.

### Cobalt enrichment of ThsB complex

His_6_-tagged ThsB1 or ThsB2 were each expressed with the complementary 3× Flag-tagged ThsB. After expression of the ThsB proteins with 1 mM IPTG, cells were either infected with Φ80α-vir (MOI 10) for 20 min or ΦNM1 prophage was induced by the addition of 1 mg ml^−1^ mitomycin C for 1 h 30 min. Where indicated, the ThsB proteins were coexpressed with Mhp from a plasmid. The resulting cells were collected by centrifugation and resuspended in 25 mM Tris pH 7.4, 100 mM NaCl, 10% glycerol, 2 mM β-mercaptoethanol, 5 mM MgSO_4_ with lysostaphin, DNase1 and EDTA-free protease inhibitor cocktail. The cells were lysed at 37 °C with shaking for 30 min before brief sonication. Lysates were clarified by ultracentrifugation and applied to cobalt affinity resin (~0.2 mg). After binding, the resin was washed six to ten times with lysis buffer before elution with lysis buffer containing 200 mM imidazole. Eluted proteins were visualized by SDS–PAGE and western blot using anti-His6 (Millipore; SAB2702218 (1:1000)) and anti-3× Flag (Millipore; F31665 (1:1000)) antibodies and mouse horseradish peroxidase conjugate secondary.

### Phylogenetic analysis of Thoeris from *S. aureus*

Bioinformatically predicted Thoeris systems in *S. aureus* were identified in the study by Doron et al.^18^. Unique Thoeris systems were identified by analysing the protein sequences of these predicted systems in Geneious Prime 2024.0.5.

### Statistics and reproducibility

All statistical analyses were performed using GraphPad Prism, version 9.5.1. Error bars and number of replicates for each experiment are defined in the figure legends. Comparisons between groups (wild type and mutant, partial and full Thoeris, for viral titre, gene expression, colony-forming units and NAD^+^ concentration) were analysed using unpaired parametric two-tailed *t*-tests with no corrections. Each experiment contains three biological replicates. Micrographs are representative of at least two independent experiments. No statistical methods were used to predetermine sample size. Sample sizes for all experiments were chosen on the basis of standards commonly used in the field of bacterial genetics and biochemistry, and were sufficient to ensure reproducibility and clear interpretation of results. For in vivo phage infection assays, each experiment was performed independently at least three times, with similar results observed across biological replicates. In vitro biochemical assays were repeated a minimum of two times with independently purified proteins to confirm reproducibility. The number of replicates was sufficient to support the robustness of observed phenotypes, such as phage resistance, NAD(H) depletion and complex formation.

### Reporting summary

Further information on research design is available in the Nature Portfolio Reporting Summary linked to this article.

## Supplementary information

Supplementary Information Supplementary Fig. 1 and Discussion.

Reporting Summary

Supplementary Data 1 Sequences of staphylococcal Thoeris operons analysed in this study.

Supplementary Data 2 Mass spectrometry data of gel slices from pull-down experiments, identifying the major head protein as the primary protein in the sample.

Supplementary Data 3 Bacterial strains, phages, plasmids and oligonucleotide primers used in this study.

## Data Availability

Data from this study are available from the lead contact upon request.
