# Supplementary material for: Bacterial TIR-based immune systems sense phage capsids to initiate defense
Source: Nat Microbiol. 2025 Oct 24;10(11):2892–902. doi: 10.1038/s41564-025-02150-0 (PMC12578639; doi:10.1038/s41564-025-02150-0)
Supplement: Supplementary file 1 — Supplementary Fig. 1 and Discussion. [file 41564_2025_2150_MOESM1_ESM.pdf]

nature microbiology

Supplementary information https://doi.org/10.1038/s41564-025-02150-0

Bacterial TIR-based immune systems sense phage capsids to initiate defense

In the format provided by the authors and unedited

Supplementary Figure 1

Red box indicates location of cropped image in figures

Figure 1B Serial dilution of phage spotted on RN4220

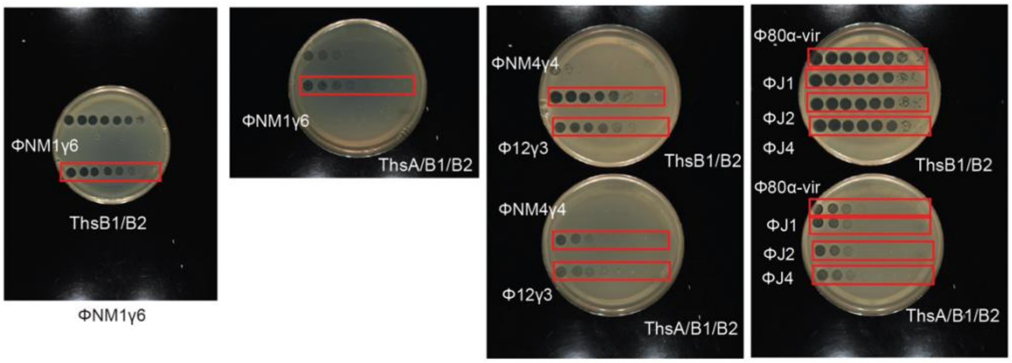

Figure 1G Time course live fluorescence microscopy of RN4220 with Φ80α-vir(::GFP)

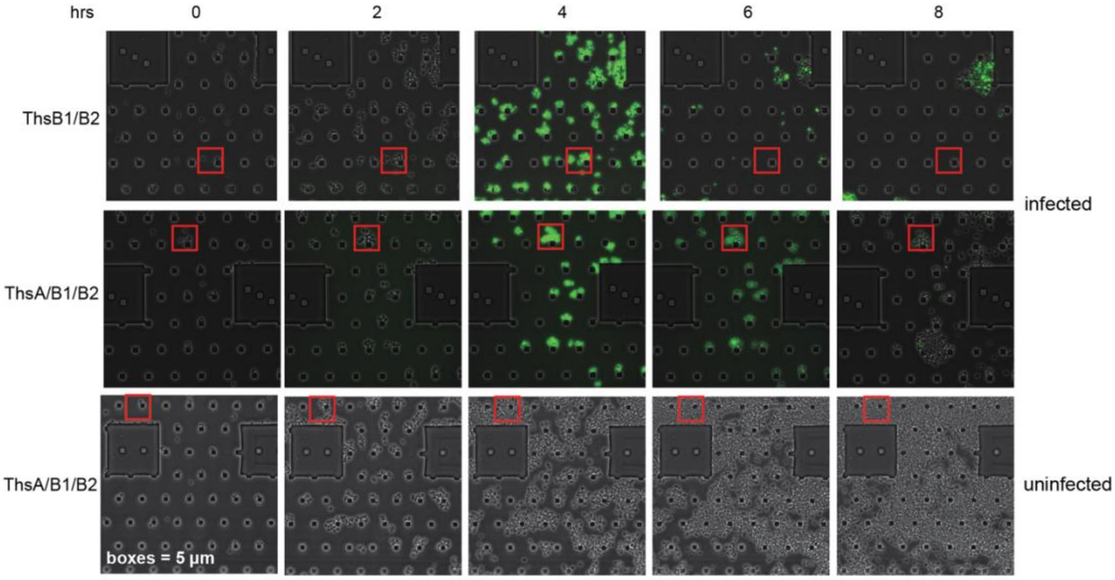

Figure 3A Western blot analysis of ThsB co-IP

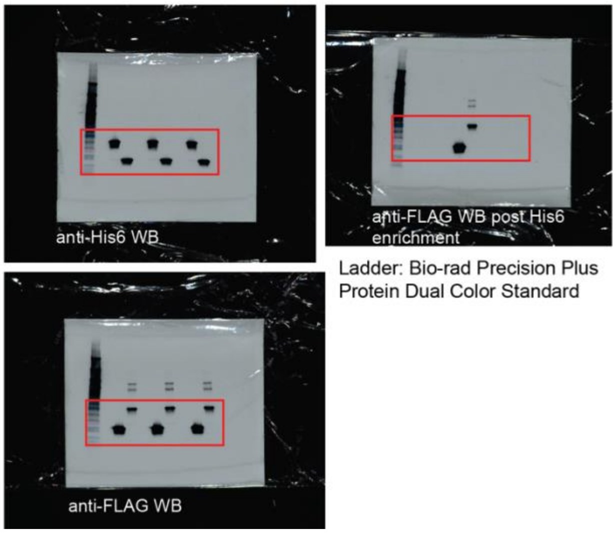

Figure 3B SDS-PAGE of ThsB co-IP

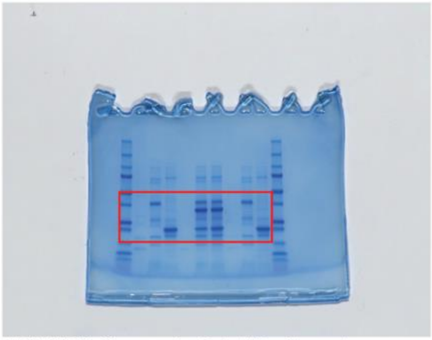

SDS-PAGE, Coomassie, Cobalt Enrichment
Ladder: Bio-rad Precision Plus `
Protein Dual Color Standard

Figure 3E Serial dilution of phage spotted on RN4220

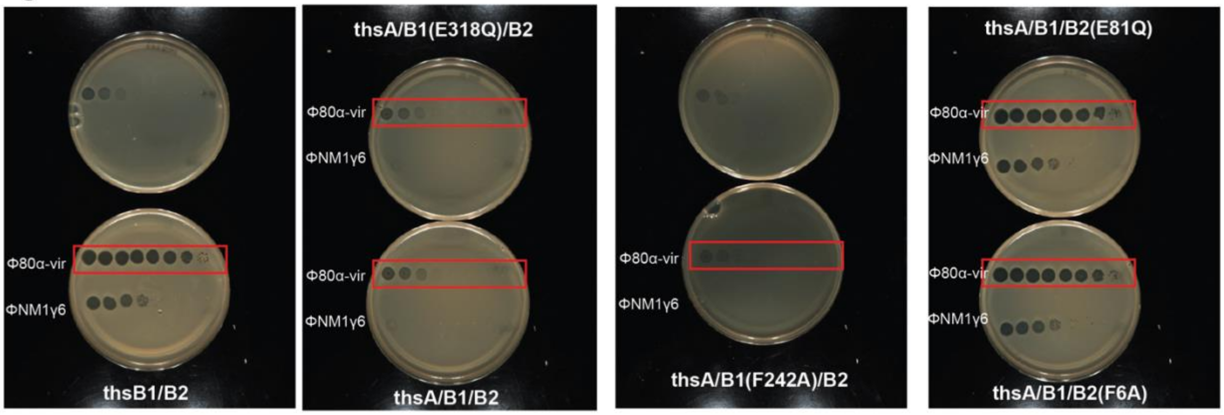

Figure 3F SDS-PAGE of ThsB co-IP

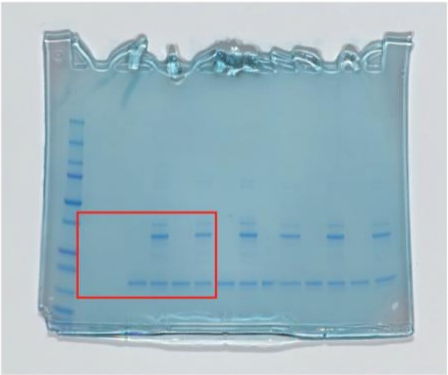

SDS-PAGE, Coomassie, Cobalt Enrichment

Figure 4A Native PAGE ThsB:Mhp complex with NAD+

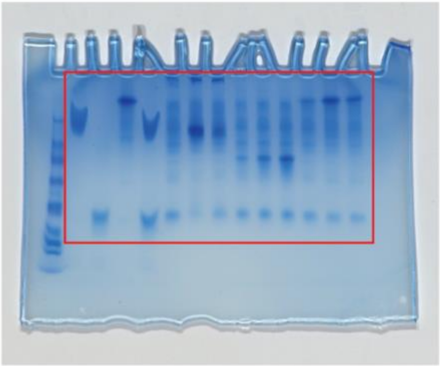

Ladder: Bio-rad Precision Plus Protein Dual Color Standard

Figure 4C FPLC analysis ThsB:Mhp fractions

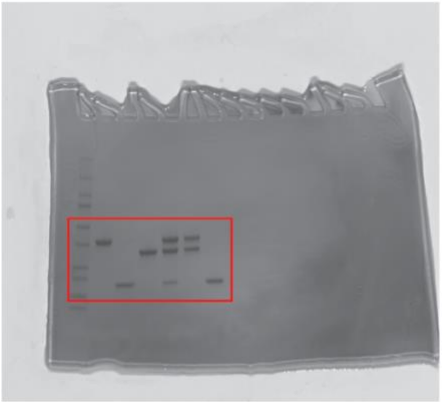

SDS-PAGE, Coomassie, FPLC fractions

Figure 4D SDS-PAGE of ThsB co-IP

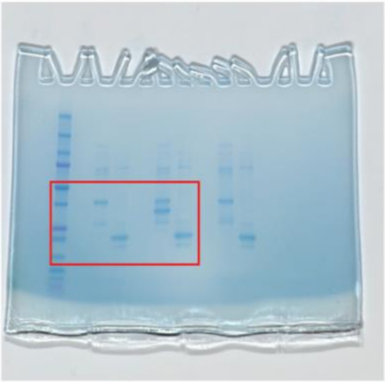

SDS-PAGE, Coomassie, Cobalt Enrichment

Figure 4E SDS-PAGE of ThsB co-IP

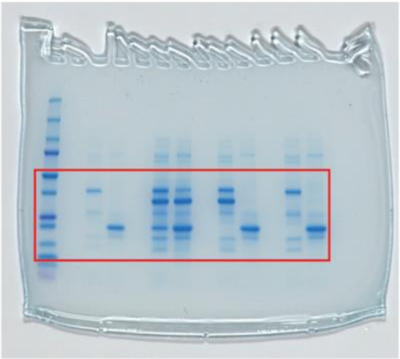

SDS-PAGE, Coomassie, Cobalt Enrichment

Ladder: Bio-rad Precision Plus Protein Dual Color Standard

Figure 6D Serial dilution of phage spotted on RN4220

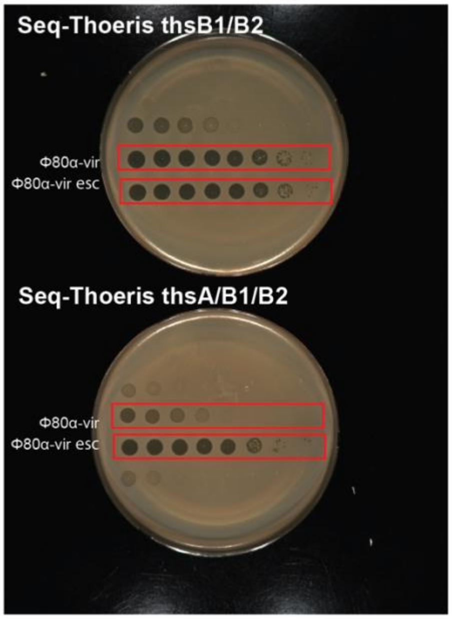

Figure 6F SDS-PAGE of ThsB co-IP from S. equinus

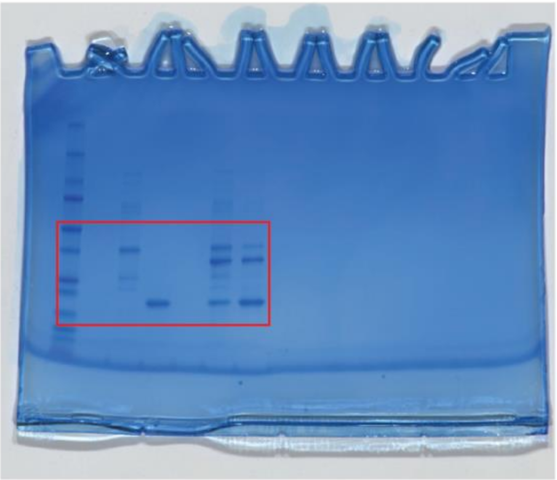

Ladder: Bio-rad Precision Plus
Protein Dual Color Standard

Extended Data Figure 1A Serial dilution of phage spotted on RN4220

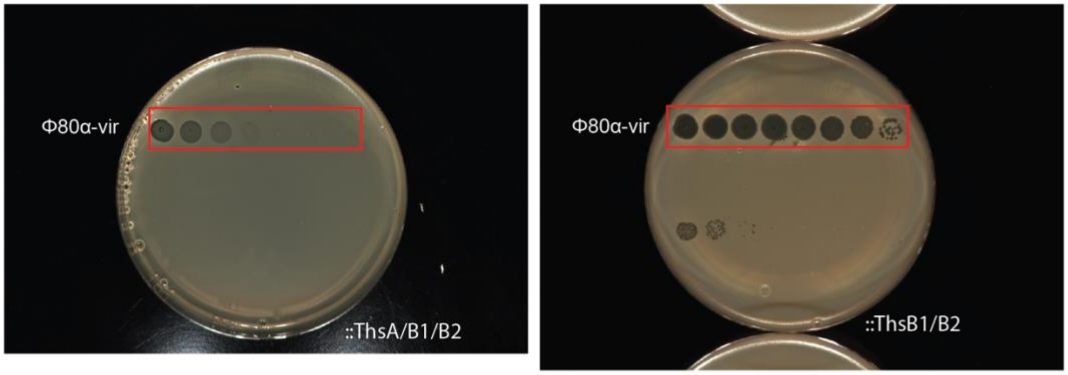

Figure Extended 2A Serial dilution of phage spotted on RN4220

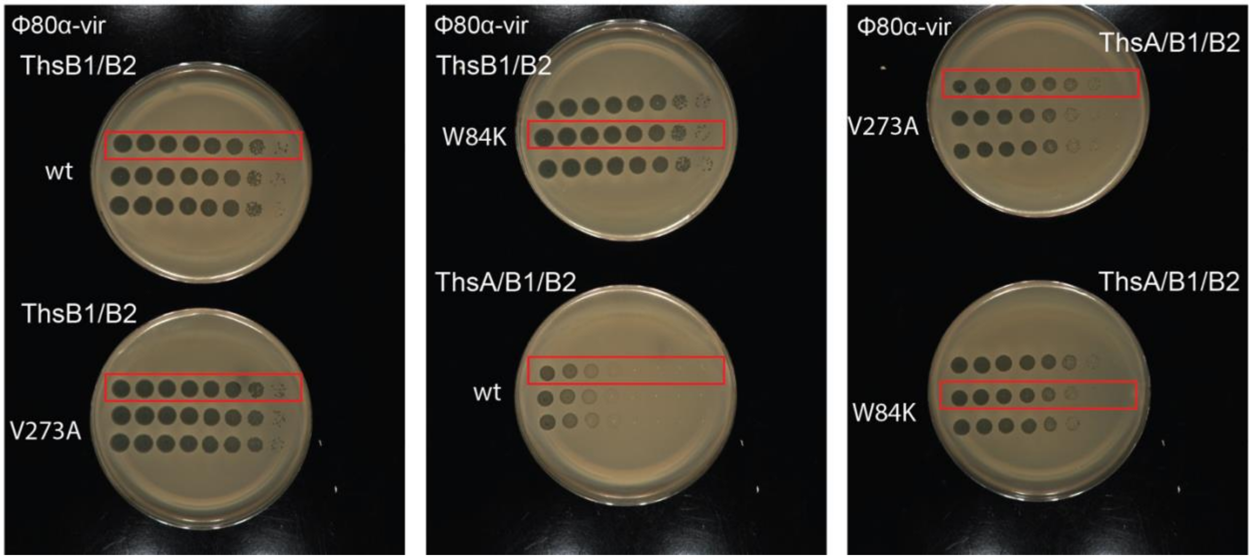

Figure Extended 2D Serial dilution of phage spotted on RN4220

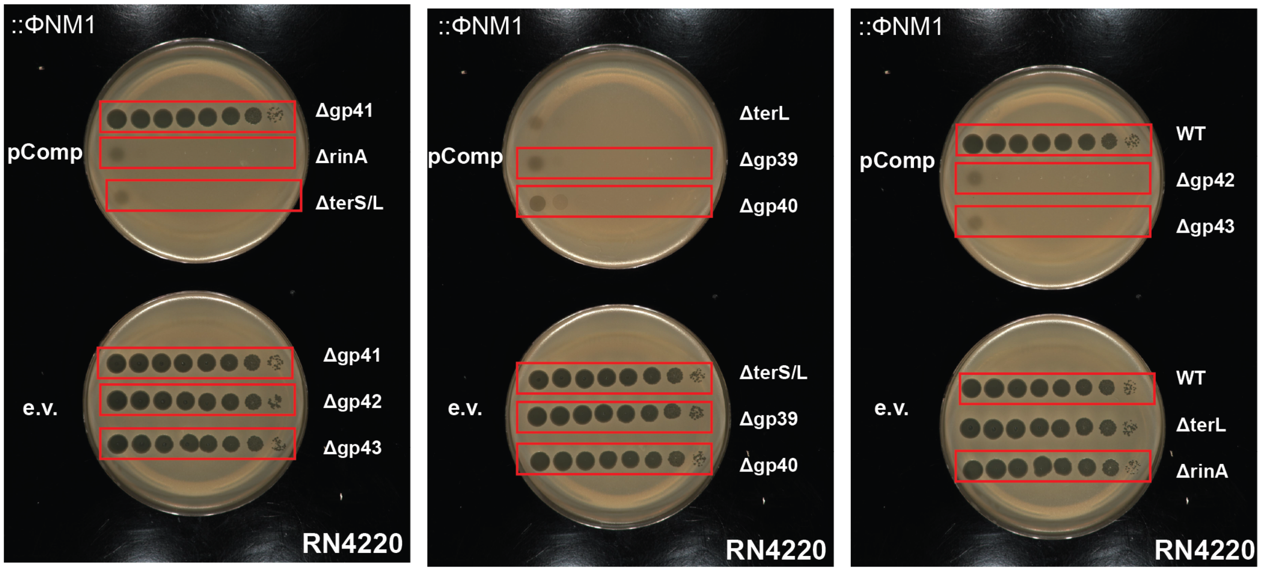

Figure Extended 3A Serial dilution of phage spotted on RN4220

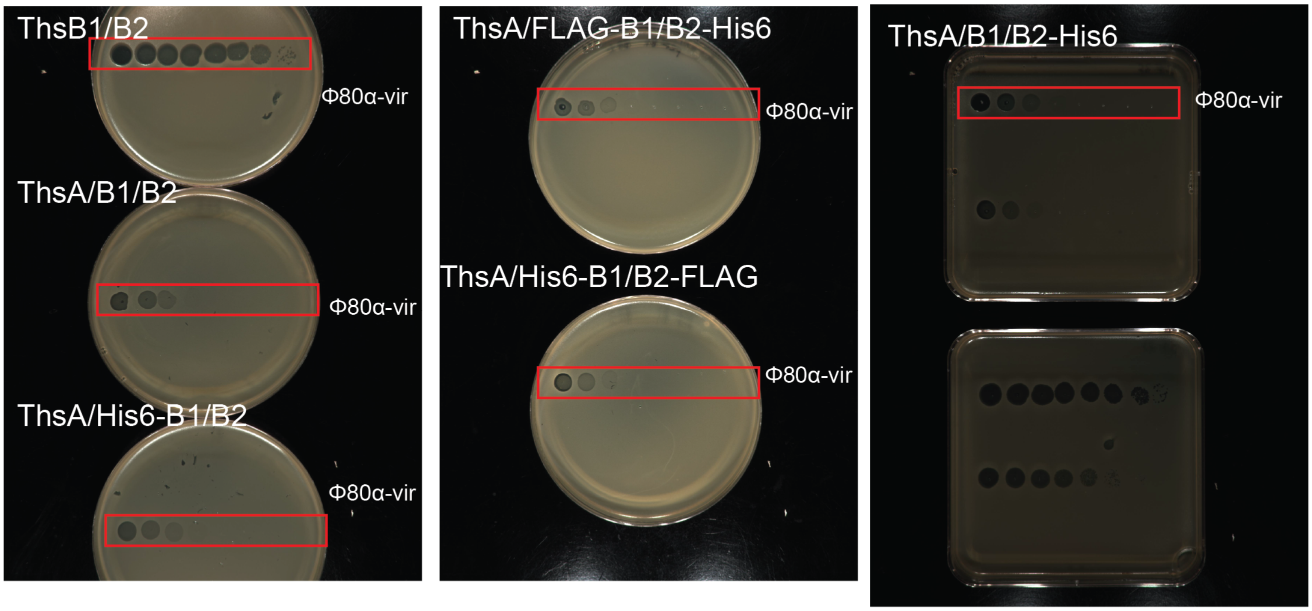

Figure Extended 5C SDS-PAGE of purified ThsA

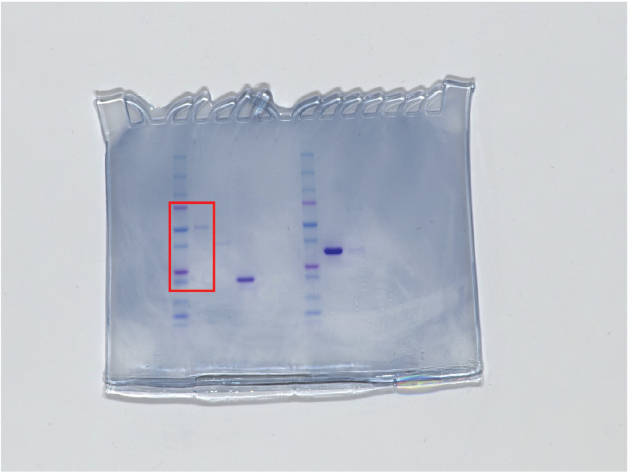

Ladder: Bio-rad Precision Plus
Protein Dual Color Standard

Figure Extended 5D SDS-PAGE of purified Mhp`WT and V273A

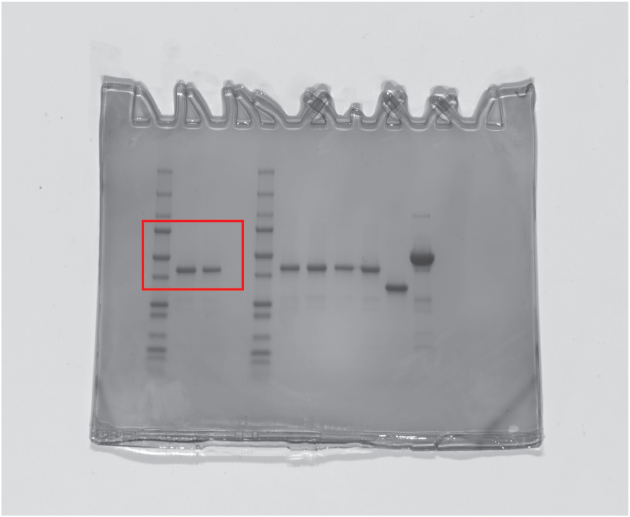

Ladder: Bio-rad Precision Plus
Protein Dual Color Standard

Figure Extended 6B SDS-PAGE of purified Mhp`

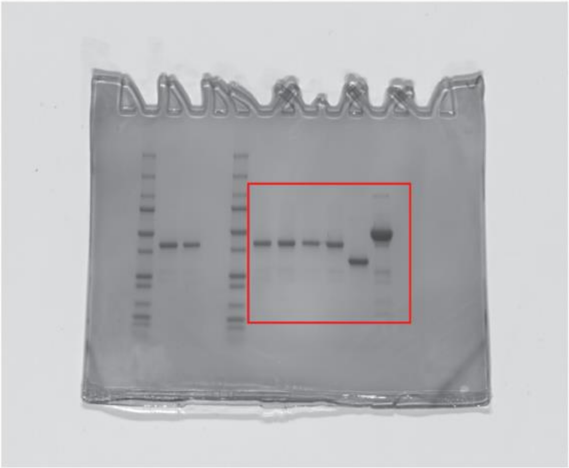

Ladder: Bio-rad Precision Plus
Protein Dual Color Standard

Extended Figure 7B/C Serial dilution of phage spotted on RN4220

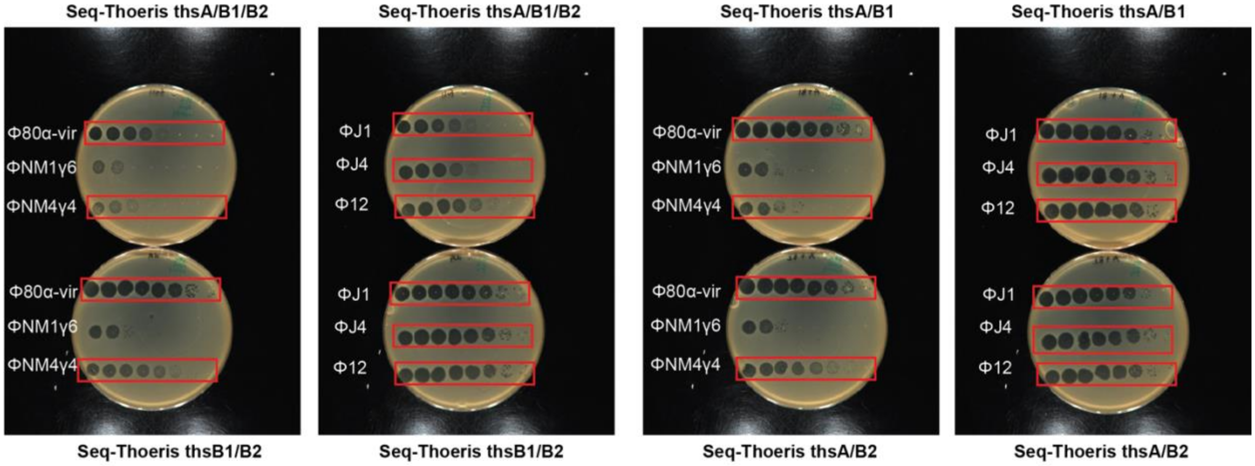

Figure Extended 7D Serial dilution of Φ80α-vir phage spotted on RN4220

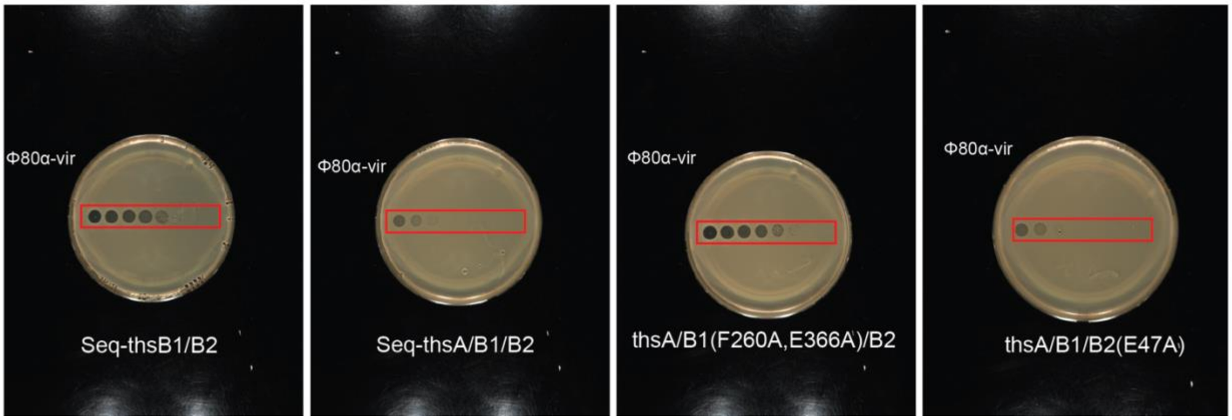

SUPPLEMENTARY DISCUSSION

for

Bacterial TIR-based immune systems sense phage capsids to initiate defence

Cameron G. Roberts[1,#*], Chloe B. Fishman[1,#], Zhiying Zhang[2], Dalton V. Banh[1], Dinshaw J. Patel[2] and Luciano A. Marraffini[1,3*]

[1]Laboratory of Bacteriology, The Rockefeller University, 1230 York Ave, New York, NY 10065, USA.

[2]Structural Biology Program, Memorial Sloan-Kettering Cancer Center, New York, NY 10065, USA

[3]Howard Hughes Medical Institute, The Rockefeller University, 1230 York Ave, New York, NY 10065, USA.

[#]these authors contributed equally to this study.

*Correspondence to: croberts@rockefeller.edu, marraffini@rockefeller.edu

Unsolved aspects of Sau-Thoeris activation

There are several features of our model that will require further investigation. For example, the structural basis of how ThsB1 binds the Mhp and how the Mhp:ThsB1 subcomplex recruits ThsB2 is not completely clear. ThsB2 does not bind ThsB1 nor Mhp alone, a result that suggests that upon formation of the Mhp:ThsB1 subcomplex, one or both of these proteins undergo a conformational change that facilitates the interaction with ThsB2. In addition, the changes in ThsB2 that trigger its cyclase activity in association with ThsB1 and Mhp remain to be determined. Since NAD^+ is required for complex formation, it is possible that ThsB2 recruitment and activation involves an interaction between its TIR domain and that of ThsB1, mediated by the substrate, a mechanism commonly observed in TIR-domain-containing immune proteins [1]. Our data indicate that mutations in the P-loop (V273A) prevent the recruitment of ThsB2 to the Mhp:ThsB1 sub-complex, and that mutations in the E-loop (W84K) abrogate all interactions between Mhp, ThsB1and ThsB2. Since these loops are 60 Å apart in the Mhp monomer, it is possible that ThsB1 associates with the E-loop region to form the Mhp:ThsB1 sub-complex and that ThsB2 binds subsequently to the P-loop region to form the final complex. Whether oligomerization of Mhp into capsid hexamers subunits is allowed and/or required for the interactions with ThsB1 and/or ThsB2 is unknown. We believe that future structural studies of the Mhp:ThsB1:ThsB2 and Mhp:ThsB1 complexes, in the presence and absence of the NAD^+ substrate, will clarify our model.

Comparison of Sau-Thoeris activation with that of other prokaryotic immune systems

Due to its functional [2] and structural [3] similarities with mammalian and plant TIR-domain

proteins that participate in the innate immune response, Thoeris is a member of a recently discovered group of anti-phage defense systems that are considered bacterial ancestors of key components of the cell-autonomous innate immune system of higher eukaryotes, which also includes CBASS (precursor of the cGAS-STING pathway) [4,5], bacterial gasdermins involved in pyroptosis [6], and prokaryotic viperins [7]. Although there is ample knowledge about these pathways in mammalian and plant cells, much less is understood about their mechanisms in bacteria and archaea, especially how they are triggered by invading phages. Prior to our work, this question was successfully answered only for two CBASS systems. One study demonstrated that staphylococcal phages produce a highly structured 400-nt RNA (cabRNA) that binds to a positively charged surface of the CdnE03 cyclase and promotes the synthesis of the cyclic dinucleotide cGAMP to initiate defense [8]. Another study found that an *Escherichia coli* CBASS system is activated by a prohead protease expressed by phage BAS13, which interacts and stimulates the EcCdnD12 cyclase to induce cell death in a CBASS-dependent manner *in vivo* [9]. Outside of the context of these ancestral defense systems, the major capsid protein of phage SECΦ27 has been found to directly activate the CapRel toxin-antitoxin system commonly present in *E. coli* prophages [10]. In addition, a number of studies have proposed, but not unequivocally demonstrated, a role for viral capsid proteins as triggers of other anti-phage responses, also in *E. coli*, such as the anti-T4 Lit protease [11], F restriction against phage T7 [12] and Pycsar immunity against T5 [13]. Finally, recent work demonstrated that, when expressed in *E. coli*, TIR-based systems such as TIR-IV and TIR-Retron recognize conserved structural features present in a diverse set of capsid, primase-helicase and tail proteins encoded by

different phages [14].

Cooperation of TIR domain-containing proteins across different domains of life

An interesting finding from our work is that bacterial TIR proteins can cooperate to provide defense. The best characterized Thoeris defense system, from *Bacillus spp.*, possesses two ThsB genes that have been shown to employ each TIR protein independently to sense different phages [2]. With the exception of the Seq-Thoeris response against ΦNM4γ4, which only requires ThsB1, our findings show an interplay between bacterial TIR proteins that is reminiscent of the interaction between TIR proteins in mammalian systems wherein TIR-domain containing sensors are activated to recruit MyD88, a TIR-domain containing adaptor protein that initiates a signaling cascade to produce an inflammatory response. For example, the IL-1 receptor (IL1R) binds cytokine IL-1α to form a complex with IL-1 receptor accessory protein (acP), which employs its TIR domain to recruit MyD88 [15]. Similarly, TLRs activated by viral nucleic acids interact with the sorting adaptor protein TIRAP (TIR domain-containing accessory protein) to recruit MyD88 through its TIR domain [16]. In all of these cases, a sensor protein (ThsB1, IL1R, TLR) recognizes a PAMP (pathogen-associated molecular pattern; Mhp, IL-1α, viral nucleic acids) and interacts with intermediate TIR-domain proteins (ThsB2, acP, TIRAP) to initiate downstream immune responses. Capsid sensing is also conserved in mammalian immunity. Analogously to ThsB1, TRIM5 is an innate immune sensor that binds to retroviral capsid lattices and both disrupts the virion structure [17,18] and initiates the TAK1 kinase signaling cascade [19] to restrict infection. Our work therefore highlights conserved strategies of cooperation between sensor and adaptor proteins and of pathogen sensing across domains of life.

References

1. Fitzgerald, K.A. & Kagan, J.C. Toll-like Receptors and the Control of Immunity. *Cell* **180**, 1044-1066 (2020).
2. Ofir, G. et al. Antiviral activity of bacterial TIR domains via immune signalling molecules. *Nature* **600**, 116-120 (2021).
3. Tamulaitiene, G. et al. Activation of Thoeris antiviral system via SIR2 effector filament assembly. *Nature* **627**, 431-436 (2024).
4. Cohen, D. et al. Cyclic GMP-AMP signalling protects bacteria against viral infection. *Nature* **574**, 691-695 (2019).
5. Morehouse, B.R. et al. STING cyclic dinucleotide sensing originated in bacteria. *Nature* **586**, 429-433 (2020).
6. Johnson, A.G. et al. Bacterial gasdermins reveal an ancient mechanism of cell death. *Science* **375**, 221-225 (2022).
7. Bernheim, A. et al. Prokaryotic viperins produce diverse antiviral molecules. *Nature* **589**, 120-124 (2021).
8. Banh, D.V. et al. Bacterial cGAS senses a viral RNA to initiate immunity. *Nature* **623**, 1001-1008 (2023).
9. Richmond-Buccola, D. et al. A large-scale type I CBASS antiphage screen identifies the phage prohead protease as a key determinant of immune activation and evasion. *Cell Host Microbe* **32**, 1074-1088 e5 (2024).
10. Zhang, T. et al. Direct activation of a bacterial innate immune system by a viral capsid protein. *Nature* **612**, 132-140 (2022).
11. Bergsland, K.J., Kao, C., Yu, Y.T., Gulati, R. & Snyder, L. A site in the T4 bacteriophage major head protein gene that can promote the inhibition of all translation in Escherichia coli. *J Mol Biol* **213**, 477-94 (1990).
12. Molineux, I.J., Schmitt, C.K. & Condreay, J.P. Mutants of bacteriophage T7 that escape F restriction. *J Mol Biol* **207**, 563-74 (1989).
13. Tal, N. et al. Cyclic CMP and cyclic UMP mediate bacterial immunity against phages. *Cell* **184**, 5728-5739 e16 (2021).
14. Wang, S. et al. The role of TIR domain-containing proteins in bacterial defense against phages. *Nat Commun* **15**, 7384 (2024).
15. Wesche, H., Henzel, W.J., Shillinglaw, W., Li, S. & Cao, Z. MyD88: an adapter that recruits IRAK to the IL-1 receptor complex. *Immunity* **7**, 837-47 (1997).
16. Bonham, K.S. et al. A promiscuous lipid-binding protein diversifies the subcellular sites of toll-like receptor signal transduction. *Cell* **156**, 705-16 (2014).
17. Stremlau, M. et al. Specific recognition and accelerated uncoating of retroviral capsids by the TRIM5alpha restriction factor. *Proc Natl Acad Sci U S A* **103**, 5514-9 (2006).
18. Zhao, G. et al. Rhesus TRIM5alpha disrupts the HIV-1 capsid at the inter-hexamer interfaces. *PLoS Pathog* **7**, e1002009 (2011).
19. Pertel, T. et al. TRIM5 is an innate immune sensor for the retrovirus capsid lattice. *Nature* **472**, 361-5 (2011).
