# Supplementary material for: Bacterial TIR-based immune systems sense phage capsids to initiate defense
Source: Nat Microbiol. 2025 Oct 24;10(11):2892–902. doi: 10.1038/s41564-025-02150-0 (PMC12578639; doi:10.1038/s41564-025-02150-0)
Supplement: Supplementary file 2 — Reporting Summary [file 41564_2025_2150_MOESM2_ESM.pdf]

nature portfolio

Corresponding author(s): Luciano A. Marraffini

Last updated by author(s): Aug 21, 2025

Reporting Summary

Nature Portfolio wishes to improve the reproducibility of the work that we publish. This form provides structure for consistency and transparency in reporting. For further information on Nature Portfolio policies, see our Editorial Policies and the Editorial Policy Checklist.

Statistics

For all statistical analyses, confirm that the following items are present in the figure legend, table legend, main text, or Methods section.

n/a	Confirmed	
☐	☒	The exact sample size (*n*) for each experimental group/condition, given as a discrete number and unit of measurement
☐	☒	A statement on whether measurements were taken from distinct samples or whether the same sample was measured repeatedly
☒	☐	The statistical test(s) used AND whether they are one- or two-sided<br>*Only common tests should be described solely by name; describe more complex techniques in the Methods section.*
☒	☐	A description of all covariates tested
☒	☐	A description of any assumptions or corrections, such as tests of normality and adjustment for multiple comparisons
☐	☒	A full description of the statistical parameters including central tendency (e.g. means) or other basic estimates (e.g. regression coefficient) AND variation (e.g. standard deviation) or associated estimates of uncertainty (e.g. confidence intervals)
☒	☐	For null hypothesis testing, the test statistic (e.g. *F*, *t*, *r*) with confidence intervals, effect sizes, degrees of freedom and *P* value noted<br>*Give P values as exact values whenever suitable.*
☒	☐	For Bayesian analysis, information on the choice of priors and Markov chain Monte Carlo settings
☒	☐	For hierarchical and complex designs, identification of the appropriate level for tests and full reporting of outcomes
☒	☐	Estimates of effect sizes (e.g. Cohen's *d*, Pearson's *r*), indicating how they were calculated

Our web collection on statistics for biologists contains articles on many of the points above.

Software and code

Policy information about availability of computer code

Data collection	N/A

Data analysis

GraphPad Prism version 9.3
PyMol version 2.5.3
SnapGene version 7.0
Adobe Photoshop 2022
Microsoft Excel
Geneious Prime version 2023.1.2
PyCharm 2020.1 (Community Edition)
Custom Python code to graph in vivo phage DNA levels from Illumina next-generation sequencing data (will be made available on GitHub upon publication or upon request)
Sickle (https://github.com/najoshi/sickle)
ABySS (https://github.com/bcgsc/abyss)
Medusa (http://combo.dbe.unifi.it/medusa)
NCBI blastn, blastp and PSI-BLAST (https://blast.ncbi.nlm.nih.gov/Blast.cgi)
HHpred (https://toolkit.tuebingen.mpg.de/tools/hhpred)
AlphaFold2 (colabfold) (https://colab.research.google.com/github/sokrypton/ColabFold/blob/main/AlphaFold2.ipynb#scrollTo=kOblAo-xetgx)
Dali server (http://ekhidna2.biocenter.helsinki.fi/dali/)
IQ-TREE 1.6.12 (http://www.iqtree.org/release/v1.6.12)
iTOL: Interactive Tree Of Life (https://itol.embl.de/)

For manuscripts utilizing custom algorithms or software that are central to the research but not yet described in published literature, software must be made available to editors and reviewers. We strongly encourage code deposition in a community repository (e.g. GitHub). See the Nature Portfolio guidelines for submitting code & software for further information.

Data

Policy information about availability of data

All manuscripts must include a data availability statement. This statement should provide the following information, where applicable:

- Accession codes, unique identifiers, or web links for publicly available datasets
- A description of any restrictions on data availability
- For clinical datasets or third party data, please ensure that the statement adheres to our policy

Lists of strains, plasmids, bacteriophages, oligonucleotides and CRISPR spacers used in this study are available in Supplementary Data File 2. Custom Python scripts used in this study will be made available on GitHub. Accession codes will be made available upon publication.

Research involving human participants, their data, or biological material

Policy information about studies with human participants or human data. See also policy information about sex, gender (identity/presentation), and sexual orientation and race, ethnicity and racism.

Reporting on sex and gender	N/A
Reporting on race, ethnicity, or other socially relevant groupings	N/A
Population characteristics	N/A
Recruitment	N/A
Ethics oversight	N/A

Note that full information on the approval of the study protocol must also be provided in the manuscript.

Field-specific reporting

Please select the one below that is the best fit for your research. If you are not sure, read the appropriate sections before making your selection.

☒ Life sciences ☐ Behavioural & social sciences ☐ Ecological, evolutionary & environmental sciences

For a reference copy of the document with all sections, see nature.com/documents/nr-reporting-summary-flat.pdf

Life sciences study design

All studies must disclose on these points even when the disclosure is negative.

Sample size	Sample sizes were not statistically predetermined, standard 3 biological replicates were used where appropriate.
Data exclusions	No data was excluded from the analysis.

Replication	All experimental findings were reliably replicated, including phage plaque assays and DNA gels where representative runs are shown.
Randomization	Not relevant to this study as there are no animal nor human experiments, and the experimental outcome does not depend on the order in which samples were analyzed in the experiments.
Blinding	Not relevant to this study as there are no animal nor human experiments, and the knowledge of the order or identity of a sample does not change the experimental outcome.

Reporting for specific materials, systems and methods

We require information from authors about some types of materials, experimental systems and methods used in many studies. Here, indicate whether each material, system or method listed is relevant to your study. If you are not sure if a list item applies to your research, read the appropriate section before selecting a response.

Materials & experimental systems

n/a	Involved in the study	
☒	☐	Antibodies
☒	☐	Eukaryotic cell lines
☒	☐	Palaeontology and archaeology
☒	☐	Animals and other organisms
☒	☐	Clinical data
☒	☐	Dual use research of concern
☒	☐	Plants

Methods

n/a	Involved in the study	
☒	☐	ChIP-seq
☒	☐	Flow cytometry
☒	☐	MRI-based neuroimaging
